# A Machine Learning Model to Improve Risk Adjustment Accuracy in Medicare

**DOI:** 10.1111/1475-6773.70093

**Published:** 2026-03-05

**Authors:** Daniel K. Shenfeld, Lindsay Warrenburg, Eli Silvert, Matthew Guido, Maggie Makar, Karen Joynt Maddox, Amol S. Navathe, Ravi Bharat Parikh, Ezekiel J. Emanuel

**Affiliations:** ^1^ Perelman School of Medicine, University of Pennsylvania Philadelphia Pennsylvania USA; ^2^ Division of Computer Science & Engineering University of Michigan Ann Arbor Michigan USA; ^3^ Washington University School of Medicine St. Louis Missouri USA; ^4^ Emory University School of Medicine Atlanta Georgia USA

**Keywords:** hierarchical condition categories, Medicare, Medicare advantage, risk adjustment

## Abstract

**Objective:**

To develop a machine learning (ML) algorithm that improves accuracy compared to the Hierarchical Condition Category (HCC) score used by the Centers for Medicare and Medicaid Services to risk‐adjust payments for > 65 million Americans.

**Study Design and Setting:**

Prognostic study using Medicare claims data to train “Franklin”, an ML algorithm predicting one‐year costs, trained using identical data to HCC. Predictive accuracy was evaluated using *R* ^2^ log cost, Spearman rho, and sensitivity and specificity.

**Data Sources and Analytic Sample:**

Random sample of 2018–2019 Part A and B claims from aged, community‐based enrollees in Traditional Medicare who were not dually eligible and did not have end‐stage renal disease.

**Principal Findings:**

The sample consisted of 4,176,666 Medicare beneficiaries (mean [SD] age 74.9 [7.2] years, 55.9% women; 85.9% Non‐Hispanic white, 5.6% African‐American, 3.4% Hispanic). Franklin was more accurate than HCC (*R* ^2^ log cost 0.44 vs. 0.15; Spearman rho 0.61 vs. 0.41, *p* < 0.001 for both). Accuracy improved for the 47% of beneficiaries with 0 HCCs and the 27% of beneficiaries with one HCC (Spearman rho 0.59 vs. 0.08 and 0.46 vs. 0.16, respectively; *p* < 0.001 for both). Franklin outperformed HCC in detecting the 20% lowest‐cost beneficiaries (sensitivity 0.60 vs. 0.34, specificity 0.90 vs. 0.83; *p* < 0.001 for both). Franklin improved accuracy over HCC for racial/ethnic minorities and rural‐dwelling beneficiaries (*R* ^2^ log cost Black 0.48 vs. 0.14, Hispanic 0.55 vs. 0.09, rural 0.36 v. 0.11; *p* < 0.001 for all), although Franklin disproportionately classified Black (15.8% vs. 10.1%) and Hispanic (22.9% vs. 12.2%) beneficiaries in the lowest predicted cost decile.

**Conclusions:**

Franklin is an ML risk adjustment model that significantly improves risk‐adjustment accuracy for Medicare beneficiaries compared to HCC. Franklin could generate improvement in payment accuracy, reduction in selection incentives, and financial savings to Medicare. Clarifying the equity impacts of more accurate risk adjustment is necessary.

**Callout Box**

- What is known on this topic
  ○ The Center for Medicare and Medicaid Services (CMS) uses Hierarchical Condition Category (HCC) scores to risk adjust payments for > 65 million Americans.
  ○ Despite frequent updates, HCC is known to be inaccurate, and CMS has explicitly called for more accurate risk adjustment methods. Yet, to date, no actual alternative model has been developed and proposed with a head‐to‐head comparison on accuracy against HCC.
- What this study adds
  ○ We developed “Franklin”, a machine learning (ML) risk adjustment model, using the same data sources and outcomes as HCC—age, sex, diagnostic codes. With the same data, the Franklin ML risk adjustment model is significantly more accurate than HCC in general and on various subpopulations and at all cost levels.
  ○ Franklin provides a concrete risk adjustment model that can be directly adopted by CMS for further evaluation and implementation.

## 1 Introduction

The Center for Medicare and Medicaid Services (CMS) has publicly emphasized the importance of developing more accurate risk adjustment to achieve more accurate and equitable payments [1]. More accurate risk adjustment mitigates payers' incentives to select healthier patients for coverage while avoiding patients who may cost more than their risk‐adjusted cost [2, 3, 4, 5, 6]. Currently, CMS employs the Hierarchical Condition Category (HCC) score to predict individuals' future one‐year costs using 3 data elements: age, sex, and diagnosis codes. In 2024, CMS used HCC to fully or partially risk‐adjust payments for at least 65 million Americans through Medicare Advantage (MA) plans (32.8 million) [7], Accountable Care Organizations (10.8 million) [8], and Affordable Care Act health exchanges (21.3 million) [9].

HCC scores are derived by grouping select diagnosis codes into categories, also called HCCs. The latest HCC version 28 (“v28”) includes 115 categories. CMS defines several demographic segments and assigns weights to each category according to the segment. The largest, “Standard” segment, consists of aged, non‐dually eligible, non‐end‐stage renal disease, community‐based, continuing enrollees and makes up 76% of all Traditional Medicare (TM) beneficiaries.

Despite frequent updates, HCC‐adjusted payments poorly predict future plan costs [10, 11]. In the Standard Segment, 47% of beneficiaries (about 12 million beneficiaries in 2023) lack a diagnosis code grouped to any HCC category, yet account for nearly one‐quarter of total Medicare spending—approximately $117 billion in 2023 [12, 13, 14]. HCC risk‐adjusts these beneficiaries based solely on age and sex, over‐predicting costs by 46% on average [15], leading to overpayment of nearly $1200 per beneficiary, or approximately $14.5 billion in total [15]. Simultaneously, HCC underpredicts costs for beneficiaries with 7 or more HCCs, disability, frailty, or high levels of social risk [15]. Collectively, these inaccuracies increase payer incentives to select for healthier individuals to enroll in their plans [2, 11, 16, 17].

The financial impact of more accurate risk adjustment algorithms depends on the specifics of the payment system. Poor design may unintentionally reinforce selection incentives, under‐treatment, and underpayment of certain subgroups such as racial and ethnic minority groups. These tradeoffs are an important context for implementation of a potential new risk adjustment model [16, 18]. Nevertheless, accuracy remains an important objective of any risk adjustment system [11]. In particular, accurate risk adjustment can mitigate favorable selection, or preferential enrollment of beneficiaries whose actual expenditures are lower than predicted. The Medicare Payment Advisory Committee (MedPAC) estimates that CMS overpaid MA plans $38 billion in 2024 due to favorable selection [19].

In an effort to promote more accurate risk adjustment, CMS has recently called for increased auditing of MA plans to curtail waste [20]. Consequently, there is interest in improving the HCC model by capturing previously uncaptured conditions [21] and more accurately predicting spending using machine learning (ML) [22]. ML models can reliably ingest patients' total diagnostic profiles and incorporate complex, non‐linear interactions among diagnosis codes to accurately predict clinical and actuarial risk.

This study's objective was to quantify and characterize improvements to the accuracy of HCC using a ML approach. We develop Franklin, an ML algorithm to predict future one‐year cost, and compare its accuracy against HCC v28. We also highlight some of the resulting tradeoffs, though the optimal design of a risk adjustment framework or how algorithms such as Franklin may be adapted to balance competing incentives is not within the scope of the present study.

To be directly comparable to HCC and easily implemented by CMS, Franklin incorporates two constraints. First, Franklin used the exact same three input variables as HCC: age, sex, and ICD‐10 diagnosis codes. However, Franklin does not use CMS's HCC categories and can score beneficiaries who lack any HCC categories. Other data elements and data sources were not considered but could be added anytime. Second, using open‐source tools and standard ML algorithms, Franklin has low computational requirements. These constraints would ease implementation of Franklin by CMS and MA plans.

## 2 Methods

This study protocol (#853407) was reviewed by the (The University of Pennsylvania) Institutional Review Board, who determined that the protocol was exempt from review.

### 2.1 Study Cohort, Covariates, and Data Preprocessing

HCC and Franklin were trained, validated, and tested on 2018 and 2019 TM claims. A random 20% sample of all Traditional (fee‐for‐service) Medicare beneficiaries in the Standard Segment was used, excluding beneficiaries with any Medicare Advantage coverage during this period. Decedents were excluded from the cohort in the primary analysis, as accounting for mortality would require statistical modeling choices that are distinct from demonstrating the value of ML for improving risk adjustment (see eFigure S1 for cohort eligibility schema).

CMS uses all Part A and B medical claims to train HCC. For Franklin, carrier, outpatient, inpatient, home health, and skilled nursing facility claims were included. Hospice and durable medical equipment claims were unavailable. Both models use demographic variables and diagnosis codes recorded in 2018 to predict total medical cost in 2019. Given the skewed distribution of costs, Franklin was trained on the logarithm of cost to generate more symmetrical distributions, a common approach when modeling healthcare costs [23, 24].

All data preprocessing, model training, and evaluation were performed using Python‐based open‐source packages (see Supplemental Methods A2 for a complete list).

### 2.2 HCC Risk Adjustment Model

Three data elements determine HCC scores: age, sex, and ICD‐10 diagnosis codes. Conceptually, HCC consists of three components:

1. An expert‐curated hierarchy of conditions (“categories”)
2. An assignment of ICD codes to the categories
3. Positive linear weights of the categories.

HCC v28 includes 115 categories. A beneficiary's HCC score is the weighted sum of categories corresponding to the recorded diagnosis codes (see Supplemental Methods B1). Scores are scaled such that 1.0 represents the average beneficiary, while scores over 1.0 represent higher‐risk beneficiaries. Payment is determined by multiplying the HCC score by a base cost, set to $12,963 in 2024 [25] and adjusted *post hoc* by regional and quality factors. These adjustments are independent of the risk score and are therefore not included in this analysis.

### 2.3 Franklin Risk Adjustment Model

**FIGURE 1:**
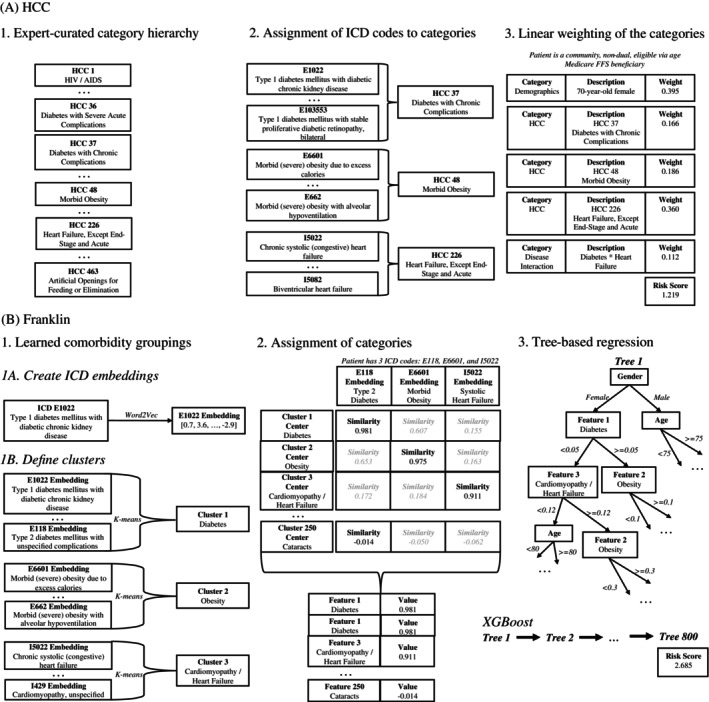
Schematic design of (A) Centers for Medicare and Medicaid Services (CMS) Hierarchical Condition Category (HCC) and (B) Franklin. *Source:* (A) HCC categories, ICD mappings, and category weights from CMS [26, 27]. The patient described in Step 3 is a theoretical example. (B) The authors' description of the high‐level modeling process. All steps show theoretical data, for illustration purposes. (A) The CMS‐HCC process involves three conceptual steps. Medical experts created a hierarchy of disease categories based on severity, called hierarchical condition categories (HCCs). Lower HCCs correspond to more severe health conditions. Each HCC is composed of a set of diagnostic codes (“ICDs”, for International Classification of Diseases). Not all ICDs are assigned to an HCC: Only those directly related to an HCC category are captured. To create a risk score for a beneficiary, each HCC is given a weight, determined by a linear regression model. Additional weights are assigned to demographic categories (age and sex) and a small number of disease interactions. (B) The Franklin process also works by creating groups of ICD codes. The groups are created in two steps. First, each ICD code is encoded as a 50‐dimensional vector called a n embedding. Geometrically close embeddings represent similar clinical codes. Second, the embeddings are grouped into 250 clusters, which are Franklin's equivalent of HCC categories. Next, each beneficiary is assigned a score for each cluster. To generate the score, the similarity is calculated between each of the beneficiary's ICD codes and the center of each cluster. The maximum similarity scores for each of the 250 clusters are the 250 diagnostic features in the model. Finally, these diagnostic features, along with age and sex, are used as the input to XGBoost, a tree‐based regression model. XGBoost's output is the beneficiary's predicted log cost. See Supporting Information for detailed discussion of methods and parameters.

Franklin uses the same data elements as HCC—age, sex, diagnosis codes—but it scores full diagnostic profiles rather than the 115 diagnostic categories. Consequently, Franklin is different from HCC in three ways. First, there is no expert‐curated hierarchy of conditions. Instead, Franklin “learns” relationships between diagnosis codes based on their co‐occurrence in claims. These relationships, learned in an unsupervised manner, are represented as high‐dimensional vectors called “embeddings.” Second, Franklin uses all diagnosis codes to create a smaller number of categories called “clusters” based on similarities among the diagnosis embeddings vectors. Like embeddings, clusters are learned in an unsupervised manner. While clusters are not assigned a label, most clusters are interpretable. For example, one cluster included five diagnosis codes directly related to obesity and another cluster included 12 codes related to abdominal pain (see Supplemental Methods B2 for more information on embeddings and clusters and their stability). A beneficiary is then assigned a value for each cluster based on their unique combination of diagnosis codes. A single diagnosis code can contribute to multiple clusters, which allows for fractional assignments across categories. The assignment of a single diagnosis code to a cluster is not fixed: its assignment is modulated by co‐occurring diagnoses, so assignments reflect a broader clinical picture rather than isolated codes. Third, HCC uses only positive linear weights to determine the risk score, while Franklin incorporates nonlinear relationships and, importantly, allows negative weights using XGBoost, a tree‐based supervised regression method. Unlike linear regression, tree‐based regression divides the data into subgroups using decision rules based on predictor values, (e.g., gender, age, cluster values), allowing for nonlinear relationships and interactions among predictors. XGBoost is an ensemble method, which means that multiple trees are used to make the final prediction for a beneficiary's risk score. Specifically, XGBoost uses a technique called boosting, where trees are built in sequence. Each tree is trained to predict the residuals of the previous trees' predictions, allowing it to gradually refine its predictions. The designs of HCC and Franklin are compared in Figure 1 and the Supplemental Methods.

To train and evaluate Franklin, the 20% sample of Traditional Medicare (TM) beneficiaries was randomly split into four cohorts: embeddings (20% of the TM sample), training (40%), validation (20%), and test (20%). The embeddings cohort was used to train the embeddings for each ICD code. The training cohort was used to create the clusters and the XGBoost model. The validation cohort was used to tune model parameters. The test cohort was used to evaluate performance metrics of Franklin compared to HCC. All the presented results were evaluated on the test set [28].

### 2.4 Measuring Risk Adjustment Accuracy

A primary objective of risk adjustment is to mitigate selection incentives by equalizing the actuarial risk incurred by an insurer when covering members with different clinical risk profiles. Conceptually, this objective can be measured by evaluating how well predicted costs track actual expenditures. This notion is sometimes referred to as “fit” in the economics literature and must be balanced against ensuring sufficient incentives to control costs [11].

Historically, fit is evaluated using an *R* ^2^ measure [29, 30]. Recent work cautions against over‐reliance on *R* ^2^, going so far as recommending that it not be reported [31]. A critical weakness of *R* ^2^ and related metrics, such as RMSE, is their extreme sensitivity to cost outliers. Even meaningful improvements to the accuracy of predictions for all but the highest cost beneficiaries may only improve *R* ^2^ marginally (see Supplemental Methods C1).

CMS uses predictive ratios (PR, ratio of predicted to observed costs) as the primary metric to evaluate HCC. When PR = 1, predicted costs match, on average, actual costs, thereby mitigating selection incentives. Predictive ratios may also be evaluated on subpopulations of interest to ensure that there is no systematic over‐ or underprediction. In particular, CMS calibrates HCC to obtain PR = 1 in each of 10 predicted risk deciles to mitigate selection between high‐ and low‐risk beneficiaries. Importantly, any risk adjustment model can be calibrated to achieve this objective.

Calibrating predictive ratios equalizes risk on average. However, due to the high variability of healthcare costs, observed PR for populations of the size of a typical MA plan may deviate substantially from 1, reflecting residual actuarial risk. Therefore, it is natural to study the rate of convergence of the PR to 1, or equivalently, its variance. In Supplemental Methods C1, we demonstrate that for log‐normal costs with log‐variance σ, the PR variance is composed of two factors. First, a structural dispersion factor $\exp\left(\sigma^{2}/2\right)$, which is independent of the risk model and reflects actuarial exposure due to cost outliers. Second, a residual dispersion factor representing variance that can be controlled by the risk model and is equal to $\sqrt{\exp\left(\left(1-R_{\log}^{2}\right)\sigma^{2}\right)-1}$, where $R_{\log}^{2}$ is *R* ^2^ on the log scale.

*R* ^2^ of log cost quantifies the reduction in variability of the predictive ratio, or equivalently, how quickly risk equalizes as population size grows. This property is of significant policy interest for three reasons. First, the residual dispersion factor represents actuarial risk and therefore selection incentives. Second, since PR variance scales inversely with population size, reducing residual dispersion will, on the margin, benefit smaller plans, helping to level the playing field. Third, from a statistical perspective, *R* ^2^ of log cost isolates cost variability due to the risk model from model‐independent structural variability due to unpredictable outliers.

### 2.5 Evaluating Performance of HCC and Franklin

Performance for HCC and Franklin predictions was evaluated against actual 2019 Medicare Parts A and B costs derived from all available claims.

The performance of Franklin versus HCC was evaluated in three ways:

1. *Statistical Model fit*. We use several statistical metrics to evaluate how well model predictions track actual costs.
  - *R* ^2^ of log cost, the percent of variance in log cost explained by the model, as discussed above. Since HCC predicts costs directly, HCC scores were recalibrated to evaluate against log cost (see Supplemental Methods C2). We note that for HCC, this calibrated *R* ^2^ of log cost is higher than *R* ^2^ of cost as reported by CMS;
  - *R* ^2^ of cost and mean absolute error (MAE) of cost, a metric less sensitive to outliers than RMSE;
  - Spearman rho, which measures the correlation between rankings of beneficiaries by predicted cost and actual cost (importantly, rankings are invariant to log transformations), and
  - the Kolmogorov–Smirnov statistic on the log scale, which tests the similarity between the distributions of predicted and actual costs and serves as an additional measure of how well the model captures the cost distribution; lower values imply better accuracy.

Each of these statistical methods of model fit has policy relevance. *R* ^2^ captures overall model fit; higher *R* ^2^ indicates that a model's predictions better match the actual cost distribution, reducing systematic over‐ or under‐payment across beneficiary groups. Spearman rho measures rank‐ordering crucial for identifying cost outliers; improved rank‐ordering particularly matters for identifying truly low‐cost beneficiaries, potentially saving CMS billions in overpayments while maintaining appropriate compensation for genuinely complex cases. The Kolmogorov–Smirnov statistic quantifies distributional alignment and has direct policy relevance: better distributional alignment means fewer beneficiaries experiencing substantial prediction errors that could incentivize cherry‐picking or lemon‐dropping behaviors.

Statistical significance was determined using a *t*‐test on 100 samples of 1000 beneficiaries each. This downsampling avoids overestimation of significance due to random fluctuations in large sample sizes.

2 *Classification performance*. Given a risk model, two binary classifiers were defined: whether a beneficiary belongs to (1) the lowest 20% of the cost distribution (< $414), and (2) the highest 20% of the cost distribution (> $6845). Similar classifiers may be defined at other cutoffs (complete threshold curves are available in eFigure D2). Three metrics were compared: (1) sensitivity, the proportion of positive cases classified correctly; (2) specificity, the proportion of negative cases classified correctly; and (3) precision, the proportion of positive predictions that were correct.
3 Frequency of substantial prediction errors, defined as the likelihood of overpredicting or underpredicting costs by ≥ 20 and ≥ 40 percentiles (complete curves in eFigure D3).

Previous evidence has suggested that risk adjustment may be unfair towards racial and ethnic minority groups [32]. To assess performance consistency, we also report performance in selected subgroups based on sex, race, ethnicity, and geography (see Supplemental Methods A2 for group definitions). Race and ethnicity were based on the enhanced race and ethnicity code that applies the Research Triangle Institute race imputation algorithm [33]. We used CMS terms for racial and ethnic groups (American Indian or Alaska Native, Asian or Other Pacific Islander, Black, Hispanic, non‐Hispanic White). To test the robustness of our model to cohort and algorithm specifications, we separately analyzed model performance (1) in a cohort that included the primary analysis cohort and decedents; and (2) when varying the high‐risk threshold to assess classification performance.

### 2.6 Comparison of Observed Costs and Measures of Actuarial Risk

CMS calibrates HCC scores to obtain PR = 1 in each of 10 risk deciles although it does not publish its methodology. A procedure to obtain predictions of observed (not log transformed) cost and calibrating predictive ratios for Franklin is described in the Supplemental Methods C2.

Cost variability within deciles reflects residual actuarial risk and incentivizes selection. We report interquartile range (IQR) and decile range (difference between 90th percentile vs. 10th percentile) of observed costs within each risk decile.

### 2.7 Financial Impact Simulations

If beneficiaries enrolled in MA plans at random, CMS expenditures would remain constant regardless of risk adjustment accuracy, because predictive ratios are calibrated to 1, the financial impact of a new risk adjustment algorithm therefore depends on downstream effects on MA plan design and enrollment. While these complex factors are not modeled here, we use reduced‐form selection simulations to gauge the magnitude of financial impact.

Favorable selection is modeled such that enrollment log‐odds depend linearly (with slope τ) on the rank difference between actual and predicted costs using both HCC and Franklin. We assume no cost‐control interventions by MA plans to isolate the impact of selection. Plan profits and financial impact for CMS are calculated by generating 10,000 samples of populations of size *N* = 20,000 from the full cohort, with a baseline MA enrollment rate of 50%. The selection strength τ is varied between 0.1 and 0.5, equivalent to enrollment probabilities of 52% to 61% for beneficiaries in the lowest decile of actual cost and highest decile of predicted cost. See Supplemental Methods C.3 for a full description of the simulation methodology and rationale.

## 3 Results

### 3.1 Population Demographics

The final cohort of 4,176,666 beneficiaries consisted of 3,890,136 (93.1%) aged and 286,530 (6.9%) disabled beneficiaries. The mean age was 74.9 years (SD 7.2), 55.9% were female, 85.9% Non‐Hispanic white, 5.6% African‐American, and 3.4% Hispanic (Table 1). Demographic characteristics were similar across all Franklin cohorts (embeddings, training, validation, testing) (See Supplemental Results D1.1).

**TABLE 1:** Demographic characteristics of the study cohort.

Number of beneficiaries	4,176,666
Sex	
Male	1,841,577 (44.09%)
Female	2,335,089 (55.91%)
Original reason for entitlement	
Aged	3,890,136 (93.14%)
Disabled (and age 65+)	286,530 (6.86%)
Age	
Mean (SD)	74.94 (7.21)
Race/ethnicity	
Non‐Hispanic White	3,588,924 (85.93%)
Black or African American	233,231 (5.58%)
Asian/Pacific Islander	80,503 (1.93%)
Hispanic	143,328 (3.43%)
American Indian/Alaska Native	15,504 (0.37%)
Other	33,475 (0.80%)
Unknown	81,701 (1.96%)

*Source:* Authors' analysis of the 20% Fee‐for‐Service Medicare cohort.

### 3.2 Performance of Franklin Versus HCC

#### 3.2.1 Model Fit

**FIGURE 2:**
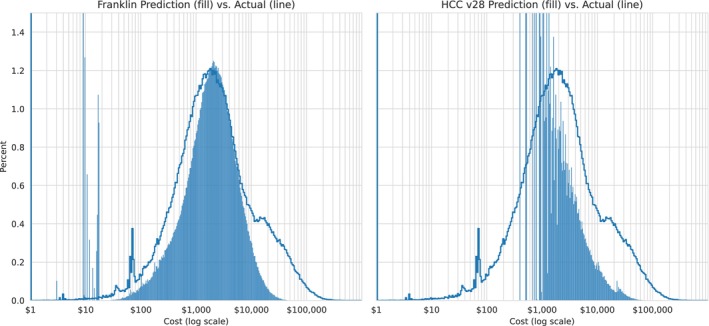
Franklin and hierarchical condition category (HCC) cost predictions compared to actual beneficiary costs. *Source:* Authors' analysis of the 20% Fee‐For‐Service Medicare cohort. Distributions of actual (solid blue line) and predicted (blue fill) 2019 medical costs for Franklin and HCC V28.

Franklin was significantly more accurate than HCC at predicting one‐year future costs (*R* ^2^ of log cost 0.44 vs. 0.15, *p* < 0.001; *R* ^2^ of cost 0.121 vs. 0.110, *p* < 0.001; Cost mean absolute error (MAE) $7223 (CI 7194–7252) vs. $7409 (CI 7379–7438), *p* < 0.001; Spearman rho 0.61 vs. 0.41, *p* < 0.001, Table 2). Figure 2 shows Franklin was significantly more accurate in capturing the observed cost distribution than HCC (Kolmogorov–Smirnov statistic 0.12 vs. 0.19, (lower statistic is more accurate) *p* < 0.001). Improvement was pronounced for the 73% of beneficiaries with either no HCCs (Spearman rho 0.59 vs. 0.08, *p* < 0.001) or 1 HCC (0.46 vs. 0.16, *p* < 0.001) (Table 2).

**TABLE 2:** Performance of Franklin and HCC across beneficiary subgroups.

		Spearman rho		*R* ^2^	
Subgroup	% (*n* = 851,441)	Franklin	HCC v28	Franklin	HCC v28
Overall					
All beneficiaries in test set	100.0	0.61	0.41	0.44	0.15
HCC count					
HCC count: 0	46.6	0.59	0.08	0.42	0.00
HCC count: 1	26.5	0.46	0.16	0.20	−0.01
HCC count: 2+	26.9	0.45	0.30	0.20	0.06
Beneficiary characteristics					
Sex: Female	55.7	0.60	0.40	0.41	0.13
Sex: Male	44.3	0.62	0.44	0.46	0.16
Age: 65–74	51.5	0.62	0.37	0.44	0.13
Age: 75–84	34.6	0.58	0.39	0.42	0.14
Age: 85+	13.9	0.52	0.38	0.38	0.13
Original Entitlement: Old age and survivor's insurance	92.4	0.61	0.41	0.44	0.15
Original Entitlement: Disability insurance benefits	7.6	0.63	0.47	0.49	0.20
Race: White	85.7	0.60	0.41	0.42	0.15
Race: Black/African American	5.7	0.65	0.45 (0.44–0.46)	0.48 (0.47–0.49)	0.14
Race: Hispanic	3.5	0.71	0.46	0.55	0.09
Density: Metropolitan^a^	76.7	0.61	0.42	0.45	0.15
Density: Micropolitan^a^	23	0.59	0.41	0.39	0.13
Density: Small town^a^	6.4	0.58	0.40	0.37	0.12
Density: Rural^a^	4.8	0.59 (0.58–0.60)	0.40 (0.39–0.41)	0.36 (0.36–0.38)	0.11
Condition: CHF^b^	1.7	0.43 (0.42–0.44)	0.34	0.20 (0.18–0.21)	0.09 (0.08–0.1)
Condition: Mental health disorders^b^	3.4	0.52	0.36	0.26	0.08 (0.08–0.10)
Condition: COPD^b^	9.6	0.51	0.40	0.26	0.12

*Note:* Bootstrapped 95% confidence intervals are ≤ 0.01 unless specified otherwise.

^a^ Metropolitan, micropolitan, small town, and rural areas were defined by rural urban commuting area (RUCA; USDA Economic Research Service, 2023).

^b^ Diabetes: HCC36, HCC37. CHF: HCC222, HCC224, HCC225. COPD: HCC280. Mental health disorders: HCC151, HCC152, HCC153, HCC154, HCC155.

*Source:* Authors' analysis of the 20% Fee‐for‐Service Medicare cohort.

#### 3.2.2 Classification Performance

Franklin significantly outperformed HCC at identifying the 20% lowest‐cost beneficiaries (sensitivity 0.60 vs. 0.35; specificity 0.90 vs. 0.84; precision 0.60 vs. 0.35, *p* < 0.001 for all). Franklin marginally outperformed HCC at identifying the 20% highest‐cost beneficiaries (sensitivity 0.47 vs. 0.44; specificity 0.87 vs. 0.86; precision 0.47 vs. 0.44; *p* < 0.001 for all). Franklin outperformed HCC in sensitivity analyses varying the classification threshold (see Supplemental Results D2.1.3).

#### 3.2.3 Frequency of Substantial Prediction Errors

Franklin was significantly less likely than HCC to overpredict medical costs by ≥ 20 percentiles (19.2% vs. 25.7%) and ≥ 40 percentiles (4.9% vs. 9.9%) or to underpredict costs by ≥ 20 percentiles (18.2% vs. 24.7%) and ≥ 40 percentiles (7.1% vs. 10.9%) (*p* < 0.001 for all) (Supplemental Results D2.2). Franklin significantly outperformed HCC with regards to over‐ and underpredictions varying the percentile threshold (see Supplemental Results D2.2).

### 3.3 Performance Among Subgroups

Franklin was significantly more accurate than HCC across beneficiaries stratified by sex (*R* ^2^ log cost females: 0.41 vs. 0.13, males: 0.46 vs. 0.16, *p* < 0.001 for both. Note: all *R* ^2^ figures reported in this section refer to *R* ^2^ of log cost), age (*R* ^2^ age 65–74: 0.44 vs. 0.13; 75–84: 0.42 vs. 0.14; 85+: 0.38 vs. 0.13, *p* < 0.001 for all). Franklin was more accurate than HCC for beneficiaries who were entitled to Medicare for either age (0.44 vs. 0.15) or disability (0.49 vs. 0.20) and for subgroups defined by race or ethnicity (white: 0.42 vs. 0.15; African‐American: 0.48 vs. 0.14; Hispanic: 0.55 vs. 0.09), geography (metropolitan: 0.45 vs. 0.15; rural: 0.36 vs. 0.11), and specific health conditions (*p* < 0.001 for all) (Table 2 and eTable D5). Sensitivity analyses including decedents were consistent with main results (Supplemental Results D.3).

Predicted cost distributions under Franklin for racial and ethnic minorities differed from HCC. Using Franklin, 15.8% of Black beneficiaries, 22.9% of Hispanics, and 8.8% of whites were in the lowest predicted cost decile, compared to 10.1%, 12.2%, and 9.6% under HCC, respectively (eTable D6 (C and D)). The redistribution of predicted cost corresponded to actual cost distributions: 16.0% of Black beneficiaries and 23.1% of Hispanics were in the lowest decile of actual cost, compared to 8.8% of white beneficiaries, whereas the distribution under HCC reflects the fact that low‐HCC beneficiaries are risk adjusted based on age and gender alone (additional fairness checks are provided in Supplemental Results D.3).

### 3.4 Comparison of Observed Costs and Measures of Actuarial Risk

Calibrated predictive ratios within risk deciles were similar between Franklin and HCC (0.97–1.02 vs. 0.97–1.01). IQR—a measure of typical cost variability, or strength of the incentive to select—was significantly lower for Franklin than HCC in all nine lower deciles (*p* < 0.001), but not the highest cost decile (Table 3). For example, Franklin's IQR was 77% lower in decile 1 than HCC (95% CI 75%–79%, $437 vs. $1881), 45% lower in decile 2 (CI 42%–49%, $1180 vs. $2153), over 20% lower in deciles 3 and 4 (CI 18%–29%), and over 10% lower in deciles 5–9 (CI lower bounds between 4% and 11%) (Table 3). Decile ranges, another measure of cost variability and propensity to select, were significantly lower for Franklin than HCC in deciles 1–2 (41% (CI 37%–46%), 73% (CI 66%–80%) respectively), but not in deciles 3–10 (eTable D7 (B)), reflecting the high variability at the top of the cost distribution.

**TABLE 3:** Predictive ratio calibration and measures of actuarial risk.

Decile	Predictive ratio		Inter‐quartile ratio		Ratio of Franklin to HCC
	Franklin	HCC	Franklin	HCC	
1	0.990	0.977	$437 ($406–$471)	$1881 ($1799–$1963)	23.2% (21.3%–25.4%)
2	0.989	0.981	$1180 ($1122–$1244)	$2153 ($2057–$2246)	54.8% (51.4%–58.5%)
3	0.991	1.026	$1609 ($1536–$1686)	$2139 ($2052–$2233)	75.2% (70.9%–79.8%)
4	0.987	1.003	$1996 ($1899–$2091)	$2587 ($2470–$2696)	77.2% (72.5%–82.1%)
5	0.998	0.995	$2461 ($2346–$2586)	$2927 ($2803–$3062)	84.1% (79.0%–89.3%)
6	1.020	0.993	$3073 ($2938–$3226)	$3514 ($3341–$3687)	87.5% (82.2%–93.3%)
7	0.988	0.996	$3989 ($3787–$4216)	$4442 ($4211–$4692)	89.8% (83.9%–96.5%)
8	0.975	0.996	$5592 ($5271–$5948)	$6526 ($6128–$6936)	85.7% (79.3%–92.8%)
9	0.995	1.006	$9122 ($8622–$9749)	$10,682 ($10,032–$11,309)	85.4% (79.6%–92.4%)
10	0.999	1.003	$21,721 ($20,694–$22,677)	$22,131 ($21,118–$23,281)	98.1% (94.0%–101.9%)

*Note:* Predictive ratios and observed cost IQRs (95% CI) for Franklin and HCC. Predictive ratios for HCC reported by CMS in table IX‐1 [34].

*Source:* Authors' analysis of the 20% Fee‐for‐Service Medicare cohort, except for Hierarchical Condition Category (HCC) predictive ratios where the source is Centers for Medicare and Medicaid Services (CMS) (table IX‐1 [34]).

### 3.5 Financial Impact Simulations

Schematic selection simulations were used to gauge the potential impact of implementing Franklin on overpayment due to favorable selection. Simulated selection strength varied between *τ* = 0.1 and 0.5, equivalent to probabilities of 52% to 61%, respectively, of enrolling a beneficiary whose risk score is in the top decile but actual cost is in the bottom decile relative to a baseline enrollment rate of 50%. For *τ* = 0.1, CMS overpayment due to favorable selection dropped from $110 (CI $106–$113) using HCC to $87 (CI $84–$90) per MA member per year (PMPY), a $23 (21%) reduction. For *τ* = 0.5, overpayment dropped from $510 (CI $507–$513) to $427 (CI $424–430) PMPY, a $99 (16%) reduction. Reduction in overpayment varied linearly for intermediate values of *τ*. For context, MedPAC estimates indicate overpayment due to favorable selection of approximately $1158 PMPY. Extrapolating over 32.8 million MA members in 2024, these figures represent annual savings of $750 million to $3.25 billion. We emphasize that these schematic simulations should be regarded as at best suggestive of the potential financial impact.

## 4 Discussion

To our knowledge, Franklin is the first published machine learning risk‐adjustment model that uses the same input data as HCC and is significantly more accurate. Franklin produces three times better model fit than HCC v28, the risk adjustment model currently used by CMS, and improvements in other measures of actuarial accuracy.

CMS uses risk‐adjustment for over 65 million Americans in MA plans, ACOs, and alternative payment models. CMS has explicitly attempted to improve risk‐adjustment for reimbursement with updated HCC versions, integrating social risk factors, and attempting to counter upcoding [35]. Yet these efforts have been inadequate. Hence, CMS has explicitly called for improvements in risk adjustment accuracy especially for more accurate spending predictions. Improving risk adjustment could lead to more accurate payments to plans and health systems, reducing the incentive to select low‐cost patients, although fairness concerns and other competing incentives must be addressed prior to implementation. Franklin realizes the explicit goal of CMS for a more accurate risk adjustment model with no added data requirements or significant computational requirements. Seven points bear emphasis.

First, Franklin significantly outperforms HCC on accuracy of cost predictions using three metrics of model fit. This improvement is the result of multiple factors. Franklin evaluates complete diagnostic profiles with nearly all ICD‐10 codes, while HCC uses a rigid hierarchy, constraining the contribution of each diagnosis to a specified value. Moreover, HCC frequently groups together disparate clinical conditions with varying cost trajectories. For example, a single HCC category (HCC23) incorporates breast cancer, melanoma, and benign neoplasms with very different clinical characteristics and cost trajectories. Furthermore, 47% of beneficiaries in the Standard Medicare Segment lack any diagnosis codes that group into an HCC category and are risk‐adjusted based solely on age and gender. Another 27% have only one HCC category. These beneficiaries represent a heterogeneous group whose diagnostic profiles often include common low‐acuity medical conditions (e.g., urinary tract infections) that contain predictive power despite not mapping to any HCC category. Franklin evaluates these beneficiaries' complete diagnostic profiles using a broader set of diagnoses codes than HCC, contributing to improved performance for 73% of beneficiaries who account for 48% of total costs in the Standard Segment, approximately $237 billion in 2023.

Second, Franklin improves model fit for the 20% highest‐cost Medicare beneficiaries. Franklin was less likely to significantly under‐ or over‐estimate costs compared to HCC. Importantly, high costs are driven in large part by inpatient admissions which cannot be predicted based solely on diagnosis codes, age, and sex [36], because only a fraction of beneficiaries with a given diagnostic profile experience an inpatient admission in any given year. This presents an inherent limitation to the accuracy of any risk adjustment model that relies solely on diagnoses, as evidenced by the much smaller improvement in accuracy metrics that are highly sensitive to outliers such as *R* ^2^ of cost. Additional data sources, such as prior utilization, laboratory, and prescription data, may help identify high‐risk beneficiaries, and could be incorporated into Franklin using a similar embedding‐based methodology. Implementation of such an approach must take care to avoid incentivizing over‐prescription and excess utilization [18, 37].

Third, Franklin's improved model fit represents an opportunity to mitigate CMS overpayment. On average, CMS overpays MA plans nearly $1200 for each of the 47% of Standard Segment beneficiaries with no HCC scores [15]. More accurately predicting the cost of these patients may allow payments to health plans to more closely reflect likely utilization of necessary services, and thereby reduce incentives to select for low‐cost but highly reimbursed beneficiaries.

Fourth, Franklin is more accurate than HCC at predicting costs for demographic subgroups such as women, racial/ethnic minorities, patients eligible due to disability, and rural‐dwelling populations. Franklin better reflects variability in actual spending distributions. For disabled beneficiaries, Franklin's superior performance compared to HCC (*R* ^2^ of log cost 0.49 vs. 0.20) suggests more equitable payments for this population who frequently face access barriers. However, there are fairness concerns that bear emphasis. In the Standard Segment, African‐American and Hispanic individuals were over‐represented in lower deciles of both the Franklin‐predicted and observed cost distributions. Compared to non‐Hispanic white populations, racial and ethnic minorities are known to have lower observed spending at similar levels of clinical risk. Furthermore, racial and ethnic minorities represent a smaller proportion of the Standard Segment compared to the overall TM population. While recently‐studied “post‐processing” methods may improve fairness in risk adjustment [38], they can be challenging to implement because of inaccuracies in race/ethnicity categorizations in Medicare data and unanticipated biases introduced by applying broad‐based correction factors to specific racial groups. Because ML‐based predictive models better reflect the observed cost‐distribution, simply applying ML models instead of HCC in the current framework may not necessarily translate into desired changes in actual payment or, more importantly, care of racial/ethnic minorities or other vulnerable subgroups. A full evaluation of the equity impacts of more accurate risk‐adjustment would encompass all Medicare segments including dual‐eligibles. To achieve a risk adjustment model that is both more equitable and more accurate, it may be necessary to incorporate covariates that better reflect spending disparities among minority populations.

Fifth, while Franklin's primary objective is to improve accuracy, its design may also limit plans' opportunities for “upcoding”—coding more intensively or recording high‐acuity codes. Upcoding costs CMS an estimated $49 billion in overpayment annually [39, 40]. Unlike HCC, which uses explicit code groupings and weights, Franklin uses complete diagnostic profiles including all available ICD‐10 codes. Under Franklin, the effect of individual ICD codes on predicted costs is non‐linear, variable, modulated by co‐occurring diagnosis codes and, importantly, could be negative. This design may make it more challenging for plans to identify select codes to meaningfully increase payments, although admittedly coding behavior may shift if Franklin were implemented.

Sixth, Franklin could easily be implemented and regularly updated by CMS and other payers. Both HCC and Franklin use diagnosis code data, so there are no additional data requirements for CMS. Franklin deliberately relies on common, public ML methods and programs with low computational requirements. There is little evidence that more computationally intensive methods, including deep learning, outperform tree‐based methods such as Franklin for administrative data [41]. Furthermore, Franklin can easily and quickly be trained to predict cost for other Medicare segments such as dual‐eligible beneficiaries, and be regularly retrained to ensure high performance over time. Importantly, while the practical and technical requirements associated with implementing Franklin are low, any implementation must be carefully designed and account for the equity considerations and payment system incentives. Best practices including periodic retraining and monitoring for data drift should be followed. Given the annual nature of MA contracts, annual retraining and recalibration would be appropriate. Drift monitoring should focus on calibration between subgroups and service types. To ensure transparency, CMS may provide an inference interface for risk score calculation or make the full model publicly available, similarly to HCC.

Finally, Franklin's accuracy gains could substantially reduce the $38 billion in favorable selection identified by MedPAC [19], while ensuring plans are appropriately compensated for the members they actually serve. By reducing the spread of observed costs within each risk decile, Franklin would lower financial risk and, correspondingly, provide less incentive for plans to select low‐cost patients within each decile. Schematic simulations indicate potential annual savings in the billions. While these results are suggestive, they also reveal inherent limitations of CMS's current risk adjustment policy that may have been obscured by the poor accuracy of HCC. Risk adjusting payments solely based on diagnostic profiles of TM beneficiaries, as currently implemented, limits model accuracy for high‐cost beneficiaries and forces tradeoffs with equitable payments for subgroups. Reliance on *R* ^2^ and predictive ratios as primary metrics obfuscates the structural limitations of risk adjustment models due to cost outliers, which may increase selection incentives and necessitate different policy solutions [42]. CMS currently lacks a standard framework to evaluate the impact of different risk adjustment models on payer selection incentives and overall spending. Developing such a framework would help policymakers align incentives and ensure payments go for beneficiaries who need the services.

## 5 Conclusion

Franklin is a ML risk adjustment model that is 3 times more accurate than HCC v28, CMS's current risk adjustment model. Franklin uses the same demographic variables and diagnostic codes as HCC, CMS's current risk adjustment method. Franklin maintains greater accuracy across the cost distribution and demographic subgroups. These findings suggest that Franklin could enhance payment accuracy and reduce selection incentives. Simultaneously, implementation must account for equity considerations and the broader incentives of the payment system.

## Funding

This work was supported by Schmidt Futures.

## Conflicts of Interest

D.K.S. reports personal fees from Village MD, Cleveland Clinic Foundation, Monogram Health, Cleveland Diagnostics, Biomedical Statistical Consulting, Rhino Health, Cylinder Health, Kalderos Inc., outside the submitted work in the past 3 years. E.J.E. reports travel reimbursement from Centre for Biomedical Ethics, National University of Singapore; travel to Global Innovation Forum AAHC, Washington, DC; travel reimbursement from Macalester College, St. Paul, MN; travel reimbursement from Oak CEO Summit, honorarium from Advocate Aurora Health Summit, travel reimbursement from DPharm Conference, honorarium and travel reimbursement from the University of Pittsburgh Medical Center Shadyside, Pittsburgh, PA; travel reimbursement from the University of California San Francisco, San Francisco, CA; travel reimbursement from Cain Brothers Conference, New York, NY; honorarium and travel reimbursement from Bowdoin College, Brunswick, ME; travel reimbursement from The Galien Foundation, travel reimbursement from HLTH 2022 Meeting, Las Vegas, NV; travel reimbursement from the Hawaii Medical Service Association, Honolulu, HI; travel reimbursement from the Tel Aviv University, Tel Aviv, Israel; travel reimbursement from The Suntory Foundation, Tokyo, Japan; honorarium and travel reimbursement from the Ontario Hospital Association, Ontario, Canada; Monetary prize and travel reimbursement from the University of Oklahoma, Oklahoma City, OK; travel reimbursement from The Quadrangle, Haverford, PA; lodging Lazard HC Leadership Summit, Walland, TN; travel reimbursement from HLTH 2023 Meeting, Las Vegas, NV; honorarium and travel reimbursement from Sanford Health, Sioux Falls, SD; honorarium and travel reimbursement from Health Plan Alliance, Atlanta, GA; honorarium and travel reimbursement from Emory Health Care Leaders Retreat, Greensboro, GA; honorarium and travel reimbursement from Employer Direct Health Care, Aspen, CO; travel reimbursement from the University of Virginia, Roslyn, VA; travel reimbursement from the New York Historical Society, New York, NY; lodging Amangiri Executive Retreat, Canyon Point, UT; travel reimbursement from Forerunner Conference, Sao Paulo, Brazil; travel reimbursement from BCEPS conference, Solstrand, Norway; travel reimbursement Arendaluska Meeting, Arendal, Norway; lodging Futures of Science Summit, Philadelphia, PA; travel reimbursement from The Galien Foundation New York; travel reimbursement from the Milken Institute Washington, DC. Dr. Emanuel is serving on the following: Member Board of Advisors Cellares Corp.; Advisor Notable Health; Member Advisory Board JSL Health Capital; Member Advisory Board Peterson Center on Health Care; Advisor Clarify Health Solutions; Consultant Health Care Foundry Inc.; Member Advisory Board Feel Better Ltd.; Consultant Korro/Coach AI Ltd.; Consultant Aberdeen Inc.; Member Board of Advisors Alto Pharmacy Holdings; Advisor Link Health Technologies; Advisor Nuna; Expert Advisory Member WHO COVID 19 Ethics & Governance Working Group; Advisory Board Member HIEX Health Innovation Exchange partnership sponsored by UN Geneva; Advisory Board Member Biden's Transition COVID‐19 Committee; Special Advisor to the Director General World Health Organization; Editorial Board Member Journal of the American Medical Association; Member Guideline Development Group (GDG) of the WHO Rapid Advice Guideline on the Use & Indications of Glucagon‐Like Peptide‐1 Receptor Agonists for Management of Adults Living with Obesity; Member Internal Advisory Board The Penn Parity Center; Advisor Dendro Technologies Inc. (CalmiGo). Dr. Emanuel reports investments in Sunstone Consulting and Aktivate. Dr. Emanuel reports royalties from his books and William Morris Endeavor. Dr. Emanuel reports the following grants: Hogan Lovells, Mendel Health Inc., University of Bergen, Jansen Pharmaceuticals Inc., Schmidt Futures Schwab Charitable Fund, Laura and John Arnold Foundation, Humana, University of Miami, Hawaii Medical Services Association, Patient Centered Outcomes Research Institute. Dr. Emanuel reports options for Nuna, Link Health Technologies Inc., Clarify Health Solutions, Healthcare Foundry Inc., Alto Pharmacy Holdings, Korro/Coach AI Ltd., FeelBetter LTD., Notable Health, Cellares Corp., Sunstone Consulting. R.B.P. reports grants from the National Institutes of Health, Department of Defense, Prostate Cancer Foundation, National Palliative Care Research Center, NCCN Foundation, Conquer Cancer Foundation, Humana, Emerson Collective, Schmidt Futures, Arnold Ventures, Mendel.ai, and Veterans Health Administration; personal fees and equity from GNS Healthcare, Thyme Care, and Onc.AI; personal fees from the ConcertAI, Cancer Study Group, Mendel.ai, Biofourmis, Archetype Therapeutics, CreditSuisse, G1 Therapeutics, Humana, and Nanology; honoraria from Flatiron and Medscape; has board membership (unpaid) at the Coalition to Transform Advanced Care and American Cancer Society; editor at the Journal of Clinical Oncology; and serves on a leadership consortium (unpaid) at the National Quality Forum, all outside the submitted work. K.J.M. receives research support from the National Heart, Lung, and Blood Institute (R01HL143421 and R01HL164561), National Institute of Nursing Research (U01NR020555) National Institute on Aging (R01AG089215), and National Center for Advancing Translational Sciences (UL1TR002345). She serves as an Associate Editor for the Journal of the American Medical Association (JAMA). She previously served on the Health Policy Advisory Council for the Centene Corporation (St. Louis, MO) and received research funding from Humana. A.S.N. reports grants from Hawaii Medical Service Association, grants from Commonwealth Fund, grants from Robert Wood Johnson Foundation, grants from Donaghue Foundation, grants from the Veterans Affairs Administration*, grants from Arnold Ventures, grants from United Healthcare, grants from Blue Cross Blue Shield of NC, grants from Humana, personal fees from Navvis Healthcare, personal fees from Elsevier Press, personal fees from Medicare Payment Advisory Commission, personal fees from Analysis Group, personal fees from Advocate Physician Partners, personal fees from the Federal Trade Commission, personal fees from Catholic Health Services Long Island, and equity from Clarify Health, personal fees and board membership for The Scan Group, and non‐compensated board membership for Integrated Services Inc. outside the submitted work in the past 3 years.

## Supporting information


**Appendix S1:** Supporting Information.

## Data Availability

The data that support the findings of this study are available from CMS. Restrictions apply to the availability of these data, which were used under license for this study. Data are available from the author(s) with the permission of CMS.
