# Supplementary material for: A Machine Learning Model to Improve Risk Adjustment Accuracy in Medicare
Source: Health Serv Res. 2026 Mar 5;61(2):e70093. doi: 10.1111/1475-6773.70093 (PMC12961717; doi:10.1111/1475-6773.70093)
Supplement: Supplementary file 1 — Appendix S1: Supporting Information. [file HESR-61-0-s001.docx]

**Supplementary Appendix**

This appendix has been provided by the authors to give readers additional information about their work.

**Table of Contents**

Contents

[SUPPLEMENTAL METHODS 3](#_Toc216343607)

[A. Study Cohort, Covariates, and Data Preprocessing 3](#_Toc216343608)

[A.1. Cohort definition 3](#_Toc216343609)

[A.2. Preprocessing 3](#_Toc216343610)

[eTable A1: Summary statistics of costs and log-costs of for test set 4](#_Toc216343611)

[B. Modeling Methodology 4](#_Toc216343612)

[B.1. CMS-HCC Methodology 4](#_Toc216343613)

[B.2. Franklin Methodology 5](#_Toc216343614)

[B.2.1. Embeddings 5](#_Toc216343615)

[eTable B1. Common ICD codes and their 10 nearest neighbors, as defined by cosine similarity 6](#_Toc216343616)

[B.2.2. Clusters 14](#_Toc216343617)

[eTable B2. Example of 10 of the 250 clusters and their component ICDs. 14](#_Toc216343618)

[B.2.3. Assignment 19](#_Toc216343619)

[eTable B3. A sample of distances between diagnosis codes and clusters 19](#_Toc216343620)

[B.2.4. XGBoost 20](#_Toc216343621)

[B.2.5. Inference 20](#_Toc216343622)

[C. Evaluating Performance of HCC and Franklin 20](#_Toc216343623)

[C.1.1. Rationale for evaluation metrics used by CMS and in this paper 20](#_Toc216343624)

[eFigure C1. Distribution of observed predictive ratios under log normal cost distributions for a population of size N=100,000. 22](#_Toc216343625)

[C.2. Franklin and HCC calibration 23](#_Toc216343626)

[C.3. Financial impact simulations 23](#_Toc216343627)

[SUPPLEMENTAL RESULTS 24](#_Toc216343628)

[D. Results 24](#_Toc216343629)

[D.1. Population Demographics 24](#_Toc216343630)

[D.1.1. Comparison between cohorts used for model training. 24](#_Toc216343631)

[eTable D1. Demographic characteristics of cohorts used for model training and evaluation. 24](#_Toc216343632)

[D.1.2. Distribution of costs by number of HCCs 25](#_Toc216343633)

[eTable D2. The distribution of beneficiaries and costs by the number of HCCs in the test set. 25](#_Toc216343634)

[D.2 Performance of Franklin vs. HCC 26](#_Toc216343635)

[D.2.1. Classification performance metrics 26](#_Toc216343636)

[D.2.1.1. Identification of beneficiaries in the top and bottom 20% of the cost distribution 26](#_Toc216343637)

[eTable D3. Classification metrics predicting cost for beneficiaries in the bottom and top 20% of the cost distribution. 95% Wilson Binomial proportion confidence intervals were all < 0.01. 26](#_Toc216343638)

[D.2.1.2. Confusion matrix for predicted and actual cost quintile 26](#_Toc216343639)

[eFigure D1. Cost quintile prediction confusion matrix for Franklin (left) and HCC (right), normalized within each quintile. Rows or columns may not sum to 100% due to rounding errors. 28](#_Toc216343640)

[D.2.1.3. Classification performance at other cost thresholds 28](#_Toc216343641)

[eFigure D2. Classification performance metrics of Franklin vs. HCC v28 in distinguishing high- vs. low-cost beneficiaries at different cost thresholds. The left panel in each figure shows a classification metric on the y-axis, while the right panel subtracts the “diagonal”, which is a line with slope = -1 and y-intercept = 1. The classification metrics are (A) Sensitivity; (B) Specificity; (C) Precision (Positive Predictive Value); (D) Negative Predictive Value. 28](#_Toc216343642)

[D.2.2. Over vs. Underprediction 33](#_Toc216343643)

[eTable D4: Prevalence of extreme over and under predictions with 95% Wilson Binomial proportion confidence intervals. 33](#_Toc216343644)

[eFigure D3: Prevalence of over and under predictions by model (Franklin vs. HCC v28). 33](#_Toc216343645)

[D.3. Comparison by sociodemographics 33](#_Toc216343646)

[D.3.1. Model fit 33](#_Toc216343647)

[eTable D5. Model performance, assessed with Spearman rho and R2, across various population cohorts. 95% confidence intervals evaluated using 1000 bootstrapped samples. 34](#_Toc216343648)

[D.3.2. Actual and predicted cost distribution by race 36](#_Toc216343649)

[eTable D6. Distribution of predicted and actual cost deciles by race, test set: (a) total beneficiary counts by race as recorded in the FFS beneficiary file (b) observed 2019 costs; (c) predicted Franklin costs; (d) predicted HCC costs. 37](#_Toc216343650)

[eFigure D4. Demographic parity: distribution of Franklin scores for Black and White beneficiaries 38](#_Toc216343651)

[eFigure D5. Calibration gaps: predicted vs. actual log cost bins, by race 38](#_Toc216343652)

[eTable D7. Individual fairness: FNR for predicting the top quintile of log cost, by race 39](#_Toc216343653)

[D.3.3. Decedent analysis. 39](#_Toc216343654)

[D.4. Model calibration and metrics of actuarial risk. 40](#_Toc216343655)

[eTable D8. (A) IQR and (B) decile range for observed costs in each decile of Franklin and HCC, and the ratio between them. 40](#_Toc216343656)

[D.5. Quantifying contribution of individual features to Franklin Scores. 41](#_Toc216343657)

[eFigure D6. SHAP plot of the tuned XGBoost model. 41](#_Toc216343658)

[References 43](#_Toc216343659)

# SUPPLEMENTAL METHODS

## A. Study Cohort, Covariates, and Data Preprocessing

### A.1. Cohort definition

To train Franklin, we used a random 20% sample of all fee-for-service (FFS) Medicare beneficiaries. Data from 2018 (the collection year) was used to identify covariates and train models, which predicted spending in 2019 (the payment year, as was done for HCC v28). Part A and Part B claims were used in the analysis. Hospice and durable medical equipment data were not available for modeling. All other claim types were included: carrier, outpatient, inpatient, home health, and skilled nursing facility.

To ensure full capture of all diagnosis codes and cost, we restricted the analysis to beneficiaries with 24 months of consecutive Traditional Medicare parts A and B coverage, excluding beneficiaries with any Medicare Advantage coverage. Following HCC cohort definitions, we focus on aged, non-dual, community-based beneficiaries, including those who were originally entitled to Medicare through disability. This population covers 75.8% of our dataset. Because of the lack of access to hospice claims, we excluded decedents from the analysis. Note that the requirement for 24 consecutive months of coverage already excludes most decedents. To reduce the effect of extreme outliers during training, beneficiaries whose 2019 capitated Medicare payment was higher than $300,000 were excluded from the training and validations sets (training outliers = 293, validation outliers = 130), but not from the test set (test outliers = 140). eFigure A1 shows a schema of eligibility in our cohort.

eFigure A1. Funnel of exclusion criteria from original data through analysis cohort.

### A.2. Preprocessing

Medicare Parts A and B claims were cleaned and pre-processed using The Tuva Project,^1^ an open-source claims processing package version 0.5.12. HCCs were extracted with the Python package HCCPy version 0.1.10. Beneficiary eligibility for the study is demonstrated in eFigure B1. The claims in each set were filtered for risk-adjustment eligibility based on the CPT/HCPCS encountering filtering logic used by CMS as implemented by Tuva Health. Further preprocessing included removing Z-codes – non-disease and non-injury codes – from the list of ICD codes, as these codes are not utilized in the CMS-HCC process.

The independent variables were age, sex, and ICD-10 CM codes, the same variables used in the HCC model. These variables were transformed via the steps described below.

The primary outcome for all spending models was the logarithm of one-year cost, with cost defined as total Medicare parts A and B spending for the 2019 (the same outcome used for all versions of the HCC model.^2^ To avoid logarithms of zero, a $1 cost was added to all beneficiaries. Negative total costs were replaced with zero. Cost was calculated from inpatient, outpatient, post-acute, home health, and physician spending. The mean (SD) cost was $6,981.54 ($17,187.10) and the median (IQR) was $1,726.83 ($4,358.11). Summary statistics are shown in eTable A1.

###### eTable A1: Summary statistics of costs and log-costs of for test set

| Metric | 2019 medical cost | Log of 2019 medical cost |
| --- | --- | --- |
| Median (IQR) | 1,726.83 (4,358.11) | 3.24 (0.93) |
| 0th percentile | 0 | 0 |
| 10th percentile | 105.44 | 2.02 |
| 20th percentile | 414.66 | 2.62 |
| 30th percentile | 756.07 | 2.88 |
| 40th percentile | 1,170.69 | 3.07 |
| 50th percentile | 1,726.83 | 3.24 |
| 60th percentile | 2,528.59 | 3.4 |
| 70th percentile | 3,836.21 | 3.58 |
| 80th percentile | 6,845.34 | 3.84 |
| 90th percentile | 17,996.5 | 4.26 |
| 100th percentile | 1,173,287.77 | 6.07 |

To assess model performance in historically marginalized populations, the following definitions were used:

1. RTI race codes were extracted from the Master Beneficiary Summary File (MBSF) and used to classify race and ethnicity.
2. Metropolitan, micropolitan, small town, and rural areas were defined by rural urban commuting area (RUCA; USDA Economic Research Service, 2023) using ZIP codes from the MBSF.

All model training was performed using Python version 3.10.4 and standard machine learning packages: scikit-learn version 1.4.2, Gensim version 4.3.2, XGBoost version 2.0.3.

## B. Modeling Methodology

### B.1. CMS-HCC Methodology

The CMS-HCC methodology is defined using data from two consecutive years: the collection year and payment year. Independent variables, consisting of demographics and diagnostic codes, are all derived from the collection year. Total Medicare payments in the payment year are the dependent variable. In version 28 of HCC, the collection and payment years are 2018 and 2019 respectively.

There are several steps in calculating a beneficiary’s HCC score. First, beneficiaries are assigned to one of nine demographic categories, which define the HCC coefficients. There are six categories for continuing enrollees in the community: non-dual aged, non-dual disabled, full benefit dual aged, full benefit dual disabled, partial benefit dual aged, partial benefit dual disabled. The remaining three categories are institutionalized beneficiaries, new enrollees, and C-SNP (chronic disease special needs plan) new enrollees. A new enrollee is a beneficiary with less than 12 months of coverage prior to the payment year. Dual status refers to being eligible for both Medicare and Medicaid.

Second, claims from the collection year are filtered for eligibility for risk adjustment. Eligibility is determined based on HCPCS / CPT code combinations and, for institutional claims, on bill type codes. Claim eligibility criteria are updated periodically.

Third, ICD codes in eligible claims are mapped to the HCC categories (“HCCs”), 115 in the current version 28. Some categories are further mapped to a hierarchy by severity. For example, Diabetes with Severe Acute Complications is more severe than Diabetes with Chronic Complications. If a beneficiary has ICDs that map to more than one HCC in a hierarchical group, only the most severe HCC is counted.

Fourth, each HCC category and demographic factor (age and gender) are assigned a coefficient. A small number of interaction terms, such as diabetes and heart failure, are included. Additional coefficients are added if a beneficiary has four or more HCCs. The raw risk score is the sum of all coefficients.

Finally, raw risk scores are adjusted based on the payment year to normalize costs year to year. These normalization factors and other adjustment factors change each year.

### B.2. Franklin Methodology

Franklin training consisted of four steps:

1. Generation of embeddings
2. Clustering
3. Assignment of observed ICDs to clusters
4. Regression using XGBoost.

Ablation experiments confirmed the contribution of each step to performance of the final model. The four steps are explained in detail below.

#### B.2.1. Embeddings

Each ICD code is represented as a continuous-valued high dimensional vector, also called an “embedding”. Embeddings are designed to be context preserving, i.e., nearby vectors represent ICD codes that tend to co-occur with similar conditions, as defined by those conditions’ own embeddings (but not necessarily with each other). Embeddings are learned from claims data using the word2vec model with parameters described below.^3^ eTable B2 shows some common ICD codes and their most similar embeddings.

The embeddings cohort was filtered to only include beneficiaries with at least one ICD code, as beneficiaries with no ICDs cannot provide any information to the Word2Vec algorithm, and included 804,769 beneficiaries.

Word2Vec was used to generate embeddings for each ICD code. The input “sentences” to the model were generated by shuffling the unique ICD codes of each patient in the embeddings cohort during the collection year. Finetuning resulted in the following hyperparameters: continuous bag of words (vs. skip-gram) model, a vector size of 50, a window size of 400, a minimum frequency count of 1, and 10 epochs. The seed was set to 123.

As an example, (fictional) beneficiary 123 has eligible claims with the following codes in 2019: I10, E785, I10, B20, E663. This beneficiary’s “sentence” would be “E785 I10 B20”. The ICD shuffling was done with a random generator in Python. Shuffling was performed to ensure that the embeddings were informed by the co-occurrence of ICDs across a beneficiary’s entire record, rather than by local sequences of codes. With a context window size of 400, Word2Vec considers up to 400 codes before and after the target code as context, meaning the shuffling will have a minimal impact on the embeddings, unless a beneficiary has more than 800 unique ICD codes.

eTable B1 illustrates the resulting embeddings. For a given diagnosis code, such as hypertension (high blood pressure), the embeddings with the closest similarity to the reference code are provided. For example, embeddings for high lipids, high cholesterol, atherosclerosis, and abnormal heart rhythms are all closely related to the embedding for high blood pressure.

###### eTable B1. Common ICD codes and their 10 nearest neighbors, as defined by cosine similarity

| Common ICDs | Common ICD description | Top 10 closest ICDs based on cosine similarity | Closest ICDs’ descriptions | Cosine similarity |
| --- | --- | --- | --- | --- |
| I10 | Essential (primary) hypertension | E785 | Hyperlipidemia, unspecified | 0.924 |
|  |  | E7800 | Pure hypercholesterolemia, unspecified | 0.842 |
|  |  | E782 | Mixed hyperlipidemia | 0.814 |
|  |  | E663 | Overweight | 0.643 |
|  |  | I2510 | Atherosclerotic heart disease of native coronary artery without angina pectoris | 0.643 |
|  |  | I119 | Hypertensive heart disease without heart failure | 0.635 |
|  |  | R9430 | Abnormal result of cardiovascular function study, unspecified | 0.610 |
|  |  | R9431 | Abnormal electrocardiogram [ECG] [EKG] | 0.594 |
|  |  | R0609 | Other forms of dyspnea | 0.584 |
|  |  | E7849 | Other hyperlipidemia | 0.564 |
| E785 | Hyperlipidemia, unspecified | I10 | Essential (primary) hypertension | 0.924 |
|  |  | E7800 | Pure hypercholesterolemia, unspecified | 0.910 |
|  |  | E782 | Mixed hyperlipidemia | 0.830 |
|  |  | I119 | Hypertensive heart disease without heart failure | 0.721 |
|  |  | R9430 | Abnormal result of cardiovascular function study, unspecified | 0.701 |
|  |  | I2510 | Atherosclerotic heart disease of native coronary artery without angina pectoris | 0.700 |
|  |  | E663 | Overweight | 0.681 |
|  |  | R9431 | Abnormal electrocardiogram [ECG] [EKG] | 0.680 |
|  |  | R9439 | Abnormal result of other cardiovascular function study | 0.645 |
|  |  | E7849 | Other hyperlipidemia | 0.644 |
| E119 | Type 2 diabetes mellitus without complications | E1165 | Type 2 diabetes mellitus with hyperglycemia | 0.960 |
|  |  | E118 | Type 2 diabetes mellitus with unspecified complications | 0.935 |
|  |  | E1169 | Type 2 diabetes mellitus with other specified complication | 0.928 |
|  |  | E1159 | Type 2 diabetes mellitus with other circulatory complications | 0.899 |
|  |  | E1121 | Type 2 diabetes mellitus with diabetic nephropathy | 0.876 |
|  |  | E1140 | Type 2 diabetes mellitus with diabetic neuropathy, unspecified | 0.874 |
|  |  | E1142 | Type 2 diabetes mellitus with diabetic polyneuropathy | 0.849 |
|  |  | E1100 | Type 2 diabetes mellitus with hyperosmolarity without nonketotic hyperglycemic-hyperosmolar coma (NKHHC) | 0.832 |
|  |  | E1149 | Type 2 diabetes mellitus with other diabetic neurological complication | 0.829 |
|  |  | E1141 | Type 2 diabetes mellitus with diabetic mononeuropathy | 0.817 |
| E039 | Hypothyroidism, unspecified | E559 | Vitamin D deficiency, unspecified | 0.853 |
|  |  | E038 | Other specified hypothyroidism | 0.847 |
|  |  | E079 | Disorder of thyroid, unspecified | 0.809 |
|  |  | E063 | Autoimmune thyroiditis | 0.808 |
|  |  | N951 | Menopausal and female climacteric states | 0.715 |
|  |  | E0590 | Thyrotoxicosis, unspecified without thyrotoxic crisis or storm | 0.701 |
|  |  | E282 | Polycystic ovarian syndrome | 0.693 |
|  |  | R946 | Abnormal results of thyroid function studies | 0.688 |
|  |  | M859 | Disorder of bone density and structure, unspecified | 0.679 |
|  |  | E069 | Thyroiditis, unspecified | 0.678 |
| E559 | Vitamin D deficiency, unspecified | E039 | Hypothyroidism, unspecified | 0.853 |
|  |  | R946 | Abnormal results of thyroid function studies | 0.790 |
|  |  | E038 | Other specified hypothyroidism | 0.786 |
|  |  | E538 | Deficiency of other specified B group vitamins | 0.780 |
|  |  | D518 | Other vitamin B12 deficiency anemias | 0.760 |
|  |  | E063 | Autoimmune thyroiditis | 0.758 |
|  |  | E069 | Thyroiditis, unspecified | 0.757 |
|  |  | D510 | Vitamin B12 deficiency anemia due to intrinsic factor deficiency | 0.751 |
|  |  | E079 | Disorder of thyroid, unspecified | 0.750 |
|  |  | D519 | Vitamin B12 deficiency anemia, unspecified | 0.748 |
| L570 | Actinic keratosis | L905 | Scar conditions and fibrosis of skin | 0.915 |
|  |  | L821 | Other seborrheic keratosis | 0.900 |
|  |  | L578 | Other skin changes due to chronic exposure to nonionizing radiation | 0.896 |
|  |  | D485 | Neoplasm of uncertain behavior of skin | 0.887 |
|  |  | X32XXXA | Exposure to sunlight, initial encounter | 0.859 |
|  |  | L579 | Skin changes due to chronic exposure to nonionizing radiation, unspecified | 0.850 |
|  |  | C44519 | Basal cell carcinoma of skin of other part of trunk | 0.849 |
|  |  | D492 | Neoplasm of unspecified behavior of bone, soft tissue, and skin | 0.845 |
|  |  | B079 | Viral wart, unspecified | 0.840 |
|  |  | L738 | Other specified follicular disorders | 0.835 |
| H2513 | Age-related nuclear cataract, bilateral | H25813 | Combined forms of age-related cataract, bilateral | 0.901 |
|  |  | H25093 | Other age-related incipient cataract, bilateral | 0.888 |
|  |  | H25013 | Cortical age-related cataract, bilateral | 0.724 |
|  |  | H40013 | Open angle with borderline findings, low risk, bilateral | 0.704 |
|  |  | H43393 | Other vitreous opacities, bilateral | 0.703 |
|  |  | H40003 | Preglaucoma, unspecified, bilateral | 0.687 |
|  |  | H40019 | Open angle with borderline findings, low risk, unspecified eye | 0.674 |
|  |  | H40033 | Anatomical narrow angle, bilateral | 0.665 |
|  |  | H2510 | Age-related nuclear cataract, unspecified eye | 0.651 |
|  |  | H53021 | Refractive amblyopia, right eye | 0.645 |
| J449 | Chronic obstructive pulmonary disease, unspecified | J431 | Panlobular emphysema | 0.823 |
|  |  | J440 | Chronic obstructive pulmonary disease with (acute) lower respiratory infection | 0.817 |
|  |  | J410 | Simple chronic bronchitis | 0.769 |
|  |  | J432 | Centrilobular emphysema | 0.767 |
|  |  | J438 | Other emphysema | 0.767 |
|  |  | J9610 | Chronic respiratory failure, unspecified whether with hypoxia or hypercapnia | 0.766 |
|  |  | J9611 | Chronic respiratory failure with hypoxia | 0.749 |
|  |  | F17211 | Nicotine dependence, cigarettes, in remission | 0.748 |
|  |  | J439 | Emphysema, unspecified | 0.721 |
|  |  | J42 | Unspecified chronic bronchitis | 0.711 |
| I5042 | Chronic combined systolic (congestive) and diastolic (congestive) heart failure | I5020 | Unspecified systolic (congestive) heart failure | 0.912 |
|  |  | I5022 | Chronic systolic (congestive) heart failure | 0.903 |
|  |  | I5023 | Acute on chronic systolic (congestive) heart failure | 0.844 |
|  |  | I5040 | Unspecified combined systolic (congestive) and diastolic (congestive) heart failure | 0.833 |
|  |  | I5043 | Acute on chronic combined systolic (congestive) and diastolic (congestive) heart failure | 0.820 |
|  |  | I5021 | Acute systolic (congestive) heart failure | 0.807 |
|  |  | I5041 | Acute combined systolic (congestive) and diastolic (congestive) heart failure | 0.795 |
|  |  | I429 | Cardiomyopathy, unspecified | 0.764 |
|  |  | I501 | Left ventricular failure, unspecified | 0.736 |
|  |  | I420 | Dilated cardiomyopathy | 0.735 |
| N184 | Chronic kidney disease, stage 4 (severe) | N2581 | Secondary hyperparathyroidism of renal origin | 0.873 |
|  |  | N181 | Chronic kidney disease, stage 1 | 0.842 |
|  |  | N182 | Chronic kidney disease, stage 2 (mild) | 0.821 |
|  |  | N250 | Renal osteodystrophy | 0.803 |
|  |  | D631 | Anemia in chronic kidney disease | 0.793 |
|  |  | N189 | Chronic kidney disease, unspecified | 0.771 |
|  |  | E875 | Hyperkalemia | 0.757 |
|  |  | R801 | Persistent proteinuria, unspecified | 0.708 |
|  |  | N19 | Unspecified kidney failure | 0.690 |
| I5022 | Chronic systolic (congestive) heart failure | I5042 | Chronic combined systolic (congestive) and diastolic (congestive) heart failure | 0.903 |
|  |  | I5020 | Unspecified systolic (congestive) heart failure | 0.857 |
|  |  | I5021 | Acute systolic (congestive) heart failure | 0.812 |
|  |  | I429 | Cardiomyopathy, unspecified | 0.791 |
|  |  | I5040 | Unspecified combined systolic (congestive) and diastolic (congestive) heart failure | 0.774 |
|  |  | I5023 | Acute on chronic systolic (congestive) heart failure | 0.746 |
|  |  | I5041 | Acute combined systolic (congestive) and diastolic (congestive) heart failure | 0.742 |
|  |  | I5032 | Chronic diastolic (congestive) heart failure | 0.702 |
|  |  | I420 | Dilated cardiomyopathy | 0.695 |
| J449 | Chronic obstructive pulmonary disease, unspecified | J431 | Panlobular emphysema | 0.823 |
|  |  | J440 | Chronic obstructive pulmonary disease with (acute) lower respiratory infection | 0.817 |
|  |  | J410 | Simple chronic bronchitis | 0.769 |
|  |  | J432 | Centrilobular emphysema | 0.767 |
|  |  | J438 | Other emphysema | 0.767 |
|  |  | J9610 | Chronic respiratory failure, unspecified whether with hypoxia or hypercapnia | 0.766 |
|  |  | J9611 | Chronic respiratory failure with hypoxia | 0.749 |
|  |  | F17211 | Nicotine dependence, cigarettes, in remission | 0.748 |
|  |  | J439 | Emphysema, unspecified | 0.721 |
| F329 | Major depressive disorder, single episode, unspecified | F418 | Other specified anxiety disorders | 0.925 |
|  |  | F339 | Major depressive disorder, recurrent, unspecified | 0.913 |
|  |  | F331 | Major depressive disorder, recurrent, moderate | 0.886 |
|  |  | F3289 | Other specified depressive episodes | 0.877 |
|  |  | F419 | Anxiety disorder, unspecified | 0.867 |
|  |  | F330 | Major depressive disorder, recurrent, mild | 0.862 |
|  |  | F39 | Unspecified mood [affective] disorder | 0.851 |
|  |  | F321 | Major depressive disorder, single episode, moderate | 0.850 |
|  |  | G4700 | Insomnia, unspecified | 0.840 |

#### B.2.2. Clusters

Clustering was used to define Franklin’s equivalent to the HCC hierarchy, by grouping similar ICDs together. A weighted k-means clustering algorithm was conducted on the most common ICD embeddings for beneficiaries in the training cohort. Embeddings were normalized before being passed to the k-means algorithm. The weights were determined by the frequency of ICD occurrences in the training set. Finetuning based on performance on the validation set implicated that training should be limited to the top 1,000 ICD codes and consist of 250 clusters. The seed was set to 123.

Inspection revealed that the 250 clusters retained a high degree of clinical intuition, with a few examples shown in eTable B2. As can be seen in the table, the clusters tend to make clinical sense. The Davies-Bouldin Index was 1.576, indicating acceptable clustering.

To evaluate the stability of these clusters, 100 iterations of the weighted k-means algorithm were conducted, each initialized with a different random seed. The mean Adjusted Rand Index was 0.66, indicating strong agreement in cluster assignments and strong overall stability.

Cluster membership was also assessed for a sample of conditions. Each cluster is defined by a group of ICDs. For each iteration of the weighted k-means algorithm, the “representative” cluster that contained the majority of the original codes was found. Descriptive statistics were tallied for each iteration, such as the number of the original cluster ICDs in the representative cluster and the number of new ICDs in the representative cluster.

Overall, the clusters remained stable across the 100 iterations and maintened good clinical sense. The hypertension cluster was completely stable, with the same single ICD code (I10) defining the cluster across all 100 iterations. The obesity cluster was originally defined by five ICDs (E6601, E6609, E662, E668, and E669). On average, 4.77 out of these 5 obesity-related ICD codes were grouped together within the representative cluster, showing high consistency. Only one new ICD code (G4730, sleep apnea, unspecified) appeared in the representative cluster, and it was observed in just 3 out of 100 iterations. Similar findings were found for clusters like hypothyroidism, osteoarthritis, and headaches. Some clusters, like abdominal pain, were not quite as closely clustered. Abdominal pain originally consisted of 12 ICD codes, but on average, only 8.28 of these ICDs appeared together in the representative cluster. These original 12 codes were spread across 2.44 clusters, on average, indicating that sometimes the abdominal pain codes separated into two clusters. Over the 100 iterations, 24 additional ICDs were included at least once in the representative abdominal pain cluster (e.g., R935, abnormal findings on diagnostic imaging of other abdominal regions, including retroperitoneum). Inspection of the results showed good clinical intuition, and only minor changes to the clusters across runs.

###### eTable B2. Example of 10 of the 250 clusters and their component ICDs.

| Cluster “Name” | ICD-10 code | ICD Description |
| --- | --- | --- |
| hypertension | I10 | Essential (primary) hypertension |
| fall | G8911 | Acute pain due to trauma |
|  | R296 | Repeated falls |
|  | R52 | Pain, unspecified |
|  | S300XXA | Contusion of lower back and pelvis, initial encounter |
|  | S72001A | Fracture of unspecified part of neck of right femur, initial encounter for closed fracture |
|  | S72001D | Fracture of unspecified part of neck of right femur, subsequent encounter for closed fracture with routine healing |
|  | S72141A | Displaced intertrochanteric fracture of right femur, initial encounter for closed fracture |
|  | T1490XA | Injury, unspecified, initial encounter |
|  | W06XXXA | Fall from bed, initial encounter |
|  | W1830XA | Fall on same level, unspecified, initial encounter |
|  | W1839XA | Other fall on same level, initial encounter |
|  | W19XXXA | Unspecified fall, initial encounter |
|  | W19XXXD | Unspecified fall, subsequent encounter |
|  | W19XXXS | Unspecified fall, sequela |
| abdominal pain | K5792 | Diverticulitis of intestine, part unspecified, without perforation or abscess without bleeding |
|  | R100 | Acute abdomen |
|  | R1010 | Upper abdominal pain, unspecified |
|  | R1011 | Right upper quadrant pain |
|  | R1012 | Left upper quadrant pain |
|  | R1030 | Lower abdominal pain, unspecified |
|  | R1031 | Right lower quadrant pain |
|  | R1032 | Left lower quadrant pain |
|  | R1033 | Periumbilical pain |
|  | R1084 | Generalized abdominal pain |
|  | R109 | Unspecified abdominal pain |
|  | R140 | Abdominal distension (gaseous) |
| obesity | E6601 | Morbid (severe) obesity due to excess calories |
|  | E6609 | Other obesity due to excess calories |
|  | E662 | Morbid (severe) obesity with alveolar hypoventilation |
|  | E668 | Other obesity |
|  | E669 | Obesity, unspecified |
| hypothyroidism | E031 | Congenital hypothyroidism without goiter |
|  | E034 | Atrophy of thyroid (acquired) |
|  | E038 | Other specified hypothyroidism |
|  | E039 | Hypothyroidism, unspecified |
|  | E063 | Autoimmune thyroiditis |
|  | E079 | Disorder of thyroid, unspecified |
|  | I158 | Other secondary hypertension |
| itchy skin conditions / allergic response | B001 | Herpesviral vesicular dermatitis |
|  | B009 | Herpesviral infection, unspecified |
|  | B354 | Tinea corporis |
|  | B359 | Dermatophytosis, unspecified |
|  | B372 | Candidiasis of skin and nail |
|  | B86 | Scabies |
|  | K130 | Diseases of lips |
|  | L0100 | Impetigo, unspecified |
|  | L0292 | Furuncle, unspecified |
|  | L2081 | Atopic neurodermatitis |
|  | L2089 | Other atopic dermatitis |
|  | L209 | Atopic dermatitis, unspecified |
|  | L237 | Allergic contact dermatitis due to plants, except food |
|  | L2389 | Allergic contact dermatitis due to other agents |
|  | L239 | Allergic contact dermatitis, unspecified cause |
|  | L2489 | Irritant contact dermatitis due to other agents |
|  | L255 | Unspecified contact dermatitis due to plants, except food |
|  | L259 | Unspecified contact dermatitis, unspecified cause |
|  | L270 | Generalized skin eruption due to drugs and medicaments taken internally |
|  | L282 | Other prurigo |
|  | L299 | Pruritus, unspecified |
|  | L301 | Dyshidrosis [pompholyx] |
|  | L309 | Dermatitis, unspecified |
|  | L438 | Other lichen planus |
|  | L439 | Lichen planus, unspecified |
|  | L500 | Allergic urticaria |
|  | L501 | Idiopathic urticaria |
|  | L508 | Other urticaria |
|  | L509 | Urticaria, unspecified |
|  | L649 | Androgenic alopecia, unspecified |
|  | L710 | Perioral dermatitis |
|  | R21 | Rash and other nonspecific skin eruption |
|  | S30860A | Insect bite (nonvenomous) of lower back and pelvis, initial encounter |
|  | S30861A | Insect bite (nonvenomous) of abdominal wall, initial encounter |
|  | S80861A | Insect bite (nonvenomous), right lower leg, initial encounter |
|  | S80862A | Insect bite (nonvenomous), left lower leg, initial encounter |
|  | T63441A | Toxic effect of venom of bees, accidental (unintentional), initial encounter |
|  | T7840XA | Allergy, unspecified, initial encounter |
|  | T7840XD | Allergy, unspecified, subsequent encounter |
|  | T887XXA | Unspecified adverse effect of drug or medicament, initial encounter |
|  | W57XXXA | Bitten or stung by nonvenomous insect and other nonvenomous arthropods, initial encounter |
| neurological conditions | D320 | Benign neoplasm of cerebral meninges |
|  | D329 | Benign neoplasm of meninges, unspecified |
|  | G319 | Degenerative disease of nervous system, unspecified |
|  | G35 | Multiple sclerosis |
|  | G40109 | Localization-related (focal) (partial) symptomatic epilepsy and epileptic syndromes with simple partial seizures, not intractable, without status epilepticus |
|  | G40209 | Localization-related (focal) (partial) symptomatic epilepsy and epileptic syndromes with complex partial seizures, not intractable, without status epilepticus |
|  | G40309 | Generalized idiopathic epilepsy and epileptic syndromes, not intractable, without status epilepticus |
|  | G4089 | Other seizures |
|  | G40909 | Epilepsy, unspecified, not intractable, without status epilepticus |
|  | G454 | Transient global amnesia |
|  | G9389 | Other specified disorders of brain |
|  | G939 | Disorder of brain, unspecified |
|  | I609 | Nontraumatic subarachnoid hemorrhage, unspecified |
|  | I619 | Nontraumatic intracerebral hemorrhage, unspecified |
|  | I629 | Nontraumatic intracranial hemorrhage, unspecified |
|  | I671 | Cerebral aneurysm, nonruptured |
|  | I6782 | Cerebral ischemia |
|  | R569 | Unspecified convulsions |
|  | R9089 | Other abnormal findings on diagnostic imaging of central nervous system |
|  | R930 | Abnormal findings on diagnostic imaging of skull and head, not elsewhere classified |
| headaches | G243 | Spasmodic torticollis |
|  | G43009 | Migraine without aura, not intractable, without status migrainosus |
|  | G43019 | Migraine without aura, intractable, without status migrainosus |
|  | G43109 | Migraine with aura, not intractable, without status migrainosus |
|  | G43709 | Chronic migraine without aura, not intractable, without status migrainosus |
|  | G43719 | Chronic migraine without aura, intractable, without status migrainosus |
|  | G43809 | Other migraine, not intractable, without status migrainosus |
|  | G43909 | Migraine, unspecified, not intractable, without status migrainosus |
|  | G44209 | Tension-type headache, unspecified, not intractable |
|  | G44219 | Episodic tension-type headache, not intractable |
|  | G44229 | Chronic tension-type headache, not intractable |
|  | G4489 | Other headache syndrome |
|  | G500 | Trigeminal neuralgia |
|  | M5481 | Occipital neuralgia |
| osteoarthritis | M1990 | Unspecified osteoarthritis, unspecified site |
|  | M1991 | Primary osteoarthritis, unspecified site |
| breast cancer | C50111 | Malignant neoplasm of central portion of right female breast |
|  | C50112 | Malignant neoplasm of central portion of left female breast |
|  | C50211 | Malignant neoplasm of upper-inner quadrant of right female breast |
|  | C50212 | Malignant neoplasm of upper-inner quadrant of left female breast |
|  | C50411 | Malignant neoplasm of upper-outer quadrant of right female breast |
|  | C50412 | Malignant neoplasm of upper-outer quadrant of left female breast |
|  | C50511 | Malignant neoplasm of lower-outer quadrant of right female breast |
|  | C50512 | Malignant neoplasm of lower-outer quadrant of left female breast |
|  | C50811 | Malignant neoplasm of overlapping sites of right female breast |
|  | C50812 | Malignant neoplasm of overlapping sites of left female breast |
|  | C50911 | Malignant neoplasm of unspecified site of right female breast |
|  | C50912 | Malignant neoplasm of unspecified site of left female breast |
|  | C50919 | Malignant neoplasm of unspecified site of unspecified female breast |
|  | C773 | Secondary and unspecified malignant neoplasm of axilla and upper limb lymph nodes |
|  | D0511 | Intraductal carcinoma in situ of right breast |
|  | D0512 | Intraductal carcinoma in situ of left breast |
|  | N630 | Unspecified lump in unspecified breast |
|  | N6310 | Unspecified lump in the right breast, unspecified quadrant |
|  | N6320 | Unspecified lump in the left breast, unspecified quadrant |
|  | N644 | Mastodynia |
|  | N6459 | Other signs and symptoms in breast |
|  | N6489 | Other specified disorders of breast |
|  | R232 | Flushing |
|  | R920 | Mammographic microcalcification found on diagnostic imaging of breast |
|  | R921 | Mammographic calcification found on diagnostic imaging of breast |
|  | R922 | Inconclusive mammogram |
|  | R928 | Other abnormal and inconclusive findings on diagnostic imaging of breast |

#### B.2.3. Assignment

The assignment step determines feature values for each beneficiary in the training cohort. For each cluster and given a beneficiary’s list of documented ICD codes, we calculated the distance between each ICD code to the cluster centroid. In this implementation, the minimal such distance is assigned as the beneficiary’s score for that cluster.

First, the cosine distance was taken between each normalized embedding and each of the 250 normalized cluster centroids. The assignment vector consists of 250 features obtained by taking the minimal value over each cluster. Note that a single ICD code may contribute to multiple clusters. eTable B3 illustrates this process using four named clusters (columns), with ICD codes as indices and cosine distances as values.

###### eTable B3. A sample of distances between diagnosis codes and clusters

| *ICD* | *ICD name* | *Hypertension cluster* | *Heart condition cluster* | *Lipid disorders cluster* | *Cataracts cluster* |
| --- | --- | --- | --- | --- | --- |
| I10 | Essential (primary) hypertension | 0 | 0.522 | 0.492 | 1.064 |
| E785 | Hyperlipidemia, unspecified | 0.076 | 0.48 | 0.409 | 1.042 |
| E7800 | Pure hypercholesterolemia, unspecified | 0.158 | 0.559 | 0.314 | 1.012 |
| E782 | Mixed hyperlipidemia | 0.186 | 0.65 | 0.24 | 1.029 |
| E663 | Overweight | 0.357 | 0.78 | 0.411 | 1.125 |
| **Resulting features** |  | **0** | **0.48** | **0.24** | **1.012** |

#### B.2.4. XGBoost

XGBoost^4^ has been shown to capture nonlinear relationships across a variety of use cases and is a workhorse of current machine learning methods. The predictors for the XGBoost model were the 250 features in the assignment vector, normalized age, and a binary indicator for sex. The dependent variable was the log_10_ transformed capitated Medicare payment for the year following the diagnosis data. Due to the size of the data and search space, finetuning was done sequentially (e.g., train max depth and minimum child weight, then row and column subsamples, then the regularization parameter, followed by the learning rate and number of trees). The parameter space was trained on the training set and tested on the validation set. The search space included a seed of 123, max depth (first, 3, 6, 9, 12, 15; then 5, 6, 7, 8), min child weight (1, 3, 5), row sub-sample (0.6, 0.7, 0.8, 0.9), column sub-sample (0.6, 0.7, 0.8, 0.9), alpha regularization (0.00001, 0.01, 0.1, 1, 100, 500, 1000, 5000, 10000), learning rate (0.01, 0.05, 0.1, 0.15, 0.2), number of estimators (100, 200, 300, 400, 500, 600, 700, 800, 900, 1000). The following hyperparameters were selected: 800 trees, with a maximum depth of 7, and a minimum child weight of 1. The learning rate was set to 0.1, the L1 regularization term was set to 100, and the seed was set to 123. The row and column sub-sample parameters didn’t affect performance, so the defaults of 1.0 were kept for both.

#### B.2.5. Inference

During inference, the validation and test data underwent the feature assignment and XGBoost steps. Predicted values for the log_10_ transformed cost were saved and used to calculate R^2^ and other metrics of interest.

## C. Evaluating Performance of HCC and Franklin

C.1.1. Rationale for evaluation metrics used by CMS and in this paper*.*

CMS uses two metrics to evaluate the HCC. Both are unsuitable for the purposes of this paper. First, predictive ratios (PR) are the ratio between average expected cost and average actual costs. CMS calibrates HCC so that PR=1 in every risk decile. However, any risk model can be calibrated to satisfy this requirement. Therefore, predictive ratios are not suitable for comparing performance between two models. Franklin predictions were calibrated using the procedure described in C.2.

The second metric used by CMS, R^2^, is the percent of variance in cost explained by the model. R^2^ is a very common metric. However, it is extremely sensitive to cost outliers. Diagnosis-based risk adjustment models cannot predict high costs due to admissions since only a fraction of beneficiaries with the same diagnostic profile will experience an inpatient admission in a given year. This limits the possible R^2^ for any diagnosis-based risk-adjustment model, regardless of its accuracy for the bottom 80% of the cost distribution.

In addition, R^2^ measures explained variance in absolute errors. A beneficiary whose predicted cost is $1,000 but actual cost is $10,000 (an absolute difference of $9,000) contributes as much to R^2^ as a beneficiary whose predicted cost is $100,000 but actual cost is $109,000. This contradicts the intuition of what it means to be a “high-risk” beneficiary. From a payer’s perspective, measuring risk in terms of medical loss ratio (MLR), the difference is between an MLR of 1000% and 109%.

To mitigate these problems, we used R^2^ of log cost, or the percentage of relative variance in cost explained, as a primary metric for optimization.

Another drawback of R^2^ is that the R^2^ values for a model evaluated on different populations may not be directly comparable. If the variance of costs is higher in one population versus another, R^2^, the percent of variance explained, will be lower. This makes Spearman’s rho a more robust metric in comparing actual and predicted costs, which is the other primary metric reported in the manuscript.

*C.1.2. Derivation of predictive ratio variance estimators*

First, we derive a distribution agnostic result. Consider i.i.d samples $Y_{1},\ldots,Y_{N}$ drawn from a cost distribution with mean $\mu_{Y}>0$ and finite variance, and an unbiased estimator $\hat{Y}$. The predictive ratio (PR) is given by

$$\text{P}\text{R}_{\text{N}}=\frac{\overline{\hat{Y}}}{\overline{Y}}=\frac{\sum\hat{Y_{i}}/N}{\sum Y_{i}/N}$$

By the central limit theorem, both the numerator and denominator converge in distribution to a normal distribution. Applying the delta method to the bivariate function $h\left( u,v \right)=\log\left( u/v \right)$ an easy calculation gives

$$\sqrt{N}\log{PR}_{N}\underset{\Rightarrow}{d}\mathcal{N}(0, V_{log})$$

where the convergence is in distribution and

$$V_{\log}=\frac{\mathrm{Var}(\hat{Y}-Y)}{\mu^{2}}$$

Estimating the variance from the sample variance of the residuals $s_{R}^{2}$ (and ignoring Bessel’s correction assuming large N) yields an estimator for the multiplicative confidence interval for the predictive ratio:

$$\mathrm{CI}_{1-\alpha}(PR)=PR_{N}\times\exp\left( \pm z_{1-\alpha/2}\sqrt{\frac{s_{R}^{2}}{\overline{Y}^{2}N}} \right)$$

This expression clarifies the actuarial and policy significance of common metrics such as RMSE and R^2^ as quantifying the rate of convergence of the PR.

Assume now that costs are log-normally distributed, namely $Z:=\log Y\sim\mathcal{N(}\mu_{Z},\sigma^{2})$ and let $\hat{Z}=Z+\epsilon$ be an unbiased estimator where the errors $\epsilon\sim\mathcal{N}(0, \tau^{2})$ are independent of $Z$. To obtain an unbiased estimator on the dollar scale, we apply the Duan smearing factor $q=e^{-\tau^{2}/2},$ ^5^ $\hat{Y}=e^{\hat{Z}}q$.

Then the per-observation residuals can be expressed as  $\hat{Y}-Y=e^{Z}\left( e^{\epsilon}q-1 \right)$. Note that $e^{\epsilon}q$ has mean 1 and the two factors are independent, and we have

$$Var\left( \hat{Y}-Y \right)\mathbb{=E}\left( e^{2Z} \right)\cdot Var\left( e^{\epsilon}q \right)=e^{2\mu_{Z}+2\sigma^{2}}(e^{\tau^{2}}-1)$$

Where the last equation follows from standard formulas for moments of the log-normal distribution. Finally, normalizing by $\mu_{Y}^{2}=e^{\mu_{Z}+2\sigma^{2}}$ we obtain the confidence interval

$$\mathrm{CI}_{1-\alpha}(PR)=PR_{N}\times\exp\left( \pm z_{1-\alpha/2}\sqrt{\frac{e^{\sigma^{2}}(e^{\tau^{2}}-1)}{N}} \right)$$

Note that the numerator decomposes as two factors. $e^{\sigma^{2}/2}$ is a structural dispersion factor, which is independent of the risk model $\hat{Z}$ and arises from the dispersion $\mathbb{E}\left( e^{2Z} \right)/\mathbb{E}^{2}\left( e^{Z} \right)=1+{CV(Y)}^{2}$. The coefficient of variation makes the structural susceptibility to outliers explicit.

The second factor $\sqrt{e^{\tau^{2}}-1}$ is the model-dependent residual dispersion. Using $R_{log}^{2}=1-\frac{\tau^{2}}{\sigma^{2}}$ and expanding to the first order, we can rewrite this factor as $\sqrt{e^{{\left( 1-R_{log}^{2} \right)\sigma}^{2}}-1}\approx\sigma\sqrt{1-R_{log}^{2}}$, explicating the role of R^2^ of log cost as measuring the model-dependent shrinkage in variance of the PR.

*C.1.3. Simulation study*

To illustrate these results, we sample costs from a log-normal distribution with parameters $\mu=7.138, \sigma=2.464$, which has the same mean and standard deviation as the observed cost distribution in our Medicare FFS cohort. Noisy estimates of log-cost are produced by varying $\tau$, and cost estimates are obtained by exponentiating and applying a smearing factor. For each value of $\tau$ we draw 10,000 random samples of population size N=100,000 and evaluate summary statistics of the PR distribution as a function of $R_{log}^{2}$. Results are shown in Figure C1. Note that the CI is not centered due to a second order bias term that was omitted from the derivation above and does not affect the half-width CI calculation. Note also that for even for moderate values of $R_{log}^{2}$, observed PR percentiles vary linearly as expected from the approximation above.

###### eFigure C1. Distribution of observed predictive ratios under log normal cost distributions for a population of size N=100,000.


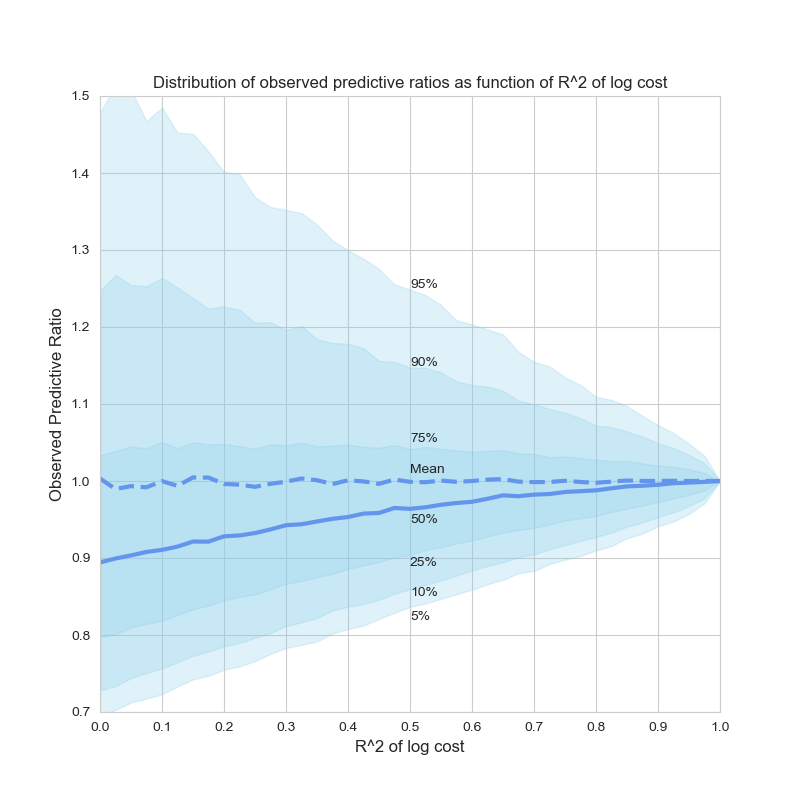


### C.2. Franklin and HCC calibration

In practice, log costs are only approximately normal and the residuals are heteroscedastic, so a global smearing factor is insufficient to obtain well-calibrated prediction on the dollar scale. To calibrate Franklin and evaluate predictive ratios, the following procedure was applied:

1. Risk percentiles were fitted to the log of mean cost at each percentile using a cubic spline.
2. Log cost predictions were converted to percentiles.
3. The spline was applied, and the results were exponentiated.

For HCC, calibration was required to evaluate performance on log cost. Here, log HCC scores were first fit to the actual cost distribution using a linear regression.

### C.3. Financial impact simulations

To assess the potential financial impact of more accurate risk adjustment due to reduced favorable selection, a reduced-form approach was taken, modeling directly the mean probability of enrollment as a function of predicted and actual costs, abstracting away the selection mechanism. Conceptually, as the distribution of actual costs among beneficiaries with similar risk scores is more concentrated, selection becomes more difficult. For the reduced-form selection model, we use cost rankings, expressed as a fraction between 0 and 1, rather than absolute costs, reflecting an assumption that selection by benefit design or reduced propensity to consume is likely to be coarse-grained.

Concretely, we model the probability *p* of beneficiary *x* to enroll in an MA plan as

$$logit\left( p(x) \right)=a+\tau\cdot(r_{pred}\left( x \right)-r_{act}\left( x \right))$$

Where *a* is the baseline enrollment log-odds (we take *a=0* reflecting a baseline enrollment probability of 50%); $\tau$represents selection strength; $r_{pred}, r_{act}$ are the predicted and actual observed (FFS) cost ranks, respectively. As the ranks vary between 0 and 1 the difference between them takes value in the interval [-1, 1].

For $\tau=0.1, 0.2, 0.3, 0.4, 0.5$, 10,000 random subsamples of 20,000 beneficiaries were generated from the full data cohort (embeddings, train, validation, and test). The full cohort was used rather than the test set to reduce resampling bias. Enrollment probabilities were generated using both HCC and Franklin, and a simulated enrolled cohort was sampled. To isolate the effect of selection, we assume no cost-reduction interventions by MA plans, so observed costs under FFS reflect actual expenditures. For each population subsample, mean predicted and actual costs under HCC and Franklin were calculated, with the difference between them reflecting overpayment attributable to favorable selection.

# SUPPLEMENTAL RESULTS

## D. Results

### D.1. Population Demographics

#### D.1.1. Comparison between cohorts used for model training.

Demographic characteristics across the four cohorts used to train and evaluate Franklin are presented in eTable D1.

###### eTable D1. Demographic characteristics of cohorts used for model training and evaluation.

|  |  | Total Population | Embedding Set | Training Set | Validation Set | Test Set |
| --- | --- | --- | --- | --- | --- | --- |
| Number of beneficiaries |  | 4,176,666^a^ | 804,769^a^ | 1,670,822 | 849,634 | 851,441 |
| Sex | Male | 1,841,577 (44.09%) | 349,730  (43.46%) | 738,258 (44.19%) | 376,253 (44.28%) | 377,336 (44.32%) |
|  | Female | 2,335,089 (55.91%) | 455,039 (56.54%) | 932,564 (55.81%) | 473,381 (55.72%) | 474,105 (55.68%) |
| Original Reason for Entitlement | Aged | 3,890,136 (93.14%) | 745,037 (92.58%) | 1,573,326  (94.16%) | 785,081  (92.40%) | 786,692 (92.40%) |
|  | Disabled  (and age 65+) | 286,530 (6.86%) | 59,732  (7.42%) | 97,496  (5.84%) | 64,553  (7.60%) | 64,749 (7.60%) |
| Age | Mean (SD) | 74.94 (7.21) | 74.98 (7.16) | 74.96 (7.23) | 74.91 (7.22) | 74.90 (7.22) |
| Race / Ethnicity | Non-Hispanic White | 3,588,924 (85.93%) | 696,362  (86.53%) | 1,433,740  (85.81%) | 728,832 (85.78%) | 729,990  (85.74%) |
|  | Black or African American | 233,231 (5.58%) | 43,406  (5.39%) | 93,383  (5.59%) | 48,137  (5.67%) | 48,305  (5.67%) |
|  | Asian / Pacific Islander | 80,503 (1.93%) | 14,646  (1.82%) | 32,726  (1.96%) | 16,517  (1.94%) | 16,614  (1.95%) |
|  | Hispanic | 143,328 (3.43%) | 25,190  (3.13%) | 58,570  (3.51%) | 29,681  (3.49%) | 29,887  (3.51%) |
|  | American Indian / Alaska Native | 15,504 (0.37%) | 2,952  (0.37%) | 6,195  (0.37%) | 3,162 (0.37%) | 3,195  (0.38%) |
|  | Other | 33,475 (0.80%) | 6,361  (0.79%) | 13,396  (0.80%) | 6,849  (0.81%) | 6,869  (0.81%) |
|  | Unknown | 81,701 (1.96%) | 15,852  (1.97%) | 32,812  (1.96%) | 16,456  (1.94%) | 16,581  (1.95%) |

^a^ The embedding cohort originally contained 849,586 beneficiaries. Of these, 804,769 had at least 1 ICD code. Since generating embeddings requires ICD codes, only beneficiaries with ICD codes were included in that analysis. With all the beneficiaries in the original embedding cohort, the total population was 4,221,483.

#### D.1.2. Distribution of costs by number of HCCs

One of the drawbacks of HCC is that nearly 75% of beneficiaries have no HCCs or a single HCC. The distribution of number of HCCs in this population is shown in eTable D2. 46.6% of the beneficiaries in the test set had no HCCs, 26.5% had one HCC, with the remaining 26.9% had two or more HCCs. The mean number of HCCs was 1.08 (SD = 1.47) and the median number of HCCs was 1.

###### eTable D2. The distribution of beneficiaries and costs by the number of HCCs in the test set.

| HCC Count | # of beneficiaries | % of beneficiaries | Mean (SD) medical cost | % of total costs |
| --- | --- | --- | --- | --- |
| 0 | 396,479 | 46.6 | 3,513 (10,575) | 23.4 |
| 1 | 225,336 | 26.5 | 6,366 (14,594) | 24.1 |
| 2 | 114,525 | 13.5 | 9,596 (19,235) | 18.5 |
| 3 | 56,205 | 6.6 | 12,960 (23,356) | 12.3 |
| 4 | 28,301 | 3.3 | 16,912 (27,490) | 8.1 |
| 5 | 14,470 | 1.7 | 21,319 (32,225) | 5.2 |
| 6 | 7,713 | 0.9 | 25,818 (36,129) | 3.4 |
| 7 | 3,998 | 0.5 | 30,880 (38,477) | 2.1 |
| 8+ | 4,414 | 0.5 | 40,659 (48,326) | 3.0 |

### D.2 Performance of Franklin vs. HCC

D.2.1. Classification performance metrics.

D.2.1.1. Identification of beneficiaries in the top and bottom 20% of the cost distribution.

The performance of Franklin and HCC version 28 can also be compared across the cost distribution. In this analysis, classification metrics are used relative to the binary outcome of the actual and predicted costs both being in the same cost quintile (bottom or top).

eTable D3 summarizes binary classification metrics for Franklin versus HCC, illustrating Franklin’s strong performance in the bottom of the cost distribution. For individuals with no HCCs, compared to the HCC model, Franklin improved sensitivity (0.64 vs. 0.30) and specificity in the bottom quintile (0.91 vs. 0.76).

###### eTable D3. Classification metrics predicting cost for beneficiaries in the bottom and top 20% of the cost distribution. 95% Wilson Binomial proportion confidence intervals were all < 0.01.

(A)

| Metric | Franklin  Bottom 20% | HCC v28 Bottom 20% | Franklin Top 20% | HCC v28 Top 20% |
| --- | --- | --- | --- | --- |
| Sensitivity | 0.60 | 0.35 | 0.47 | 0.44 |
| Specificity | 0.90 | 0.84 | 0.87 | 0.86 |
| Negative Predictive Values | 0.90 | 0.84 | 0.87 | 0.86 |
| Positive Predictive Values | 0.60 | 0.35 | 0.47 | 0.44 |

D.2.1.2. Confusion matrix for predicted and actual cost quintile.

In addition to binary classification metrics, the quintile confusion matrix may be evaluated. eFigure D1 shows the classification confusion matrix for cost quintile, i.e., the proportion of beneficiaries within each predicted cost and actual cost quintiles. The confusion matrices are normalized so that each row and each column sum to 100%. For example, 60% of beneficiaries predicted by Franklin to be in the bottom quintile have observed costs in the bottom quintile (top left value).

###### eFigure D1. Cost quintile prediction confusion matrix for Franklin (left) and HCC (right), normalized within each quintile. Rows or columns may not sum to 100% due to rounding errors.


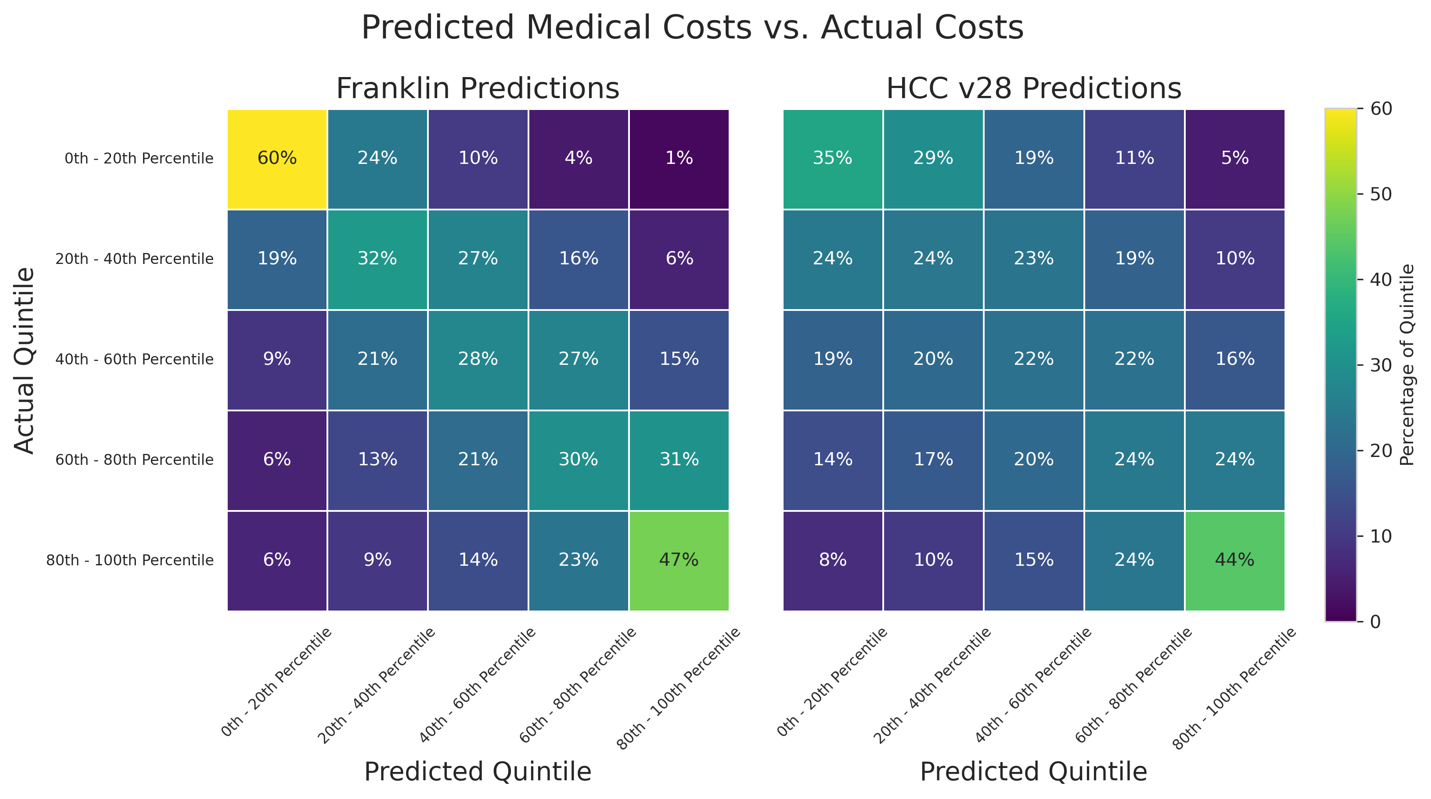


D.2.1.3. Classification performance at other cost thresholds.

eFigure D2 provides classification metrics for binary classifiers detecting whether a beneficiary’s cost falls within the bottom X%, where X is a threshold varying between 0 and 100%.

###### **eFigure D2**. Classification performance metrics of Franklin vs. HCC v28 in distinguishing high- vs. low-cost beneficiaries at different cost thresholds. The left panel in each figure shows a classification metric on the y-axis, while the right panel subtracts the “diagonal”, which is a line with slope = -1 and y-intercept = 1. The classification metrics are (A) Sensitivity; (B) Specificity; (C) Precision (Positive Predictive Value); (D) Negative Predictive Value.

A. Sensitivity


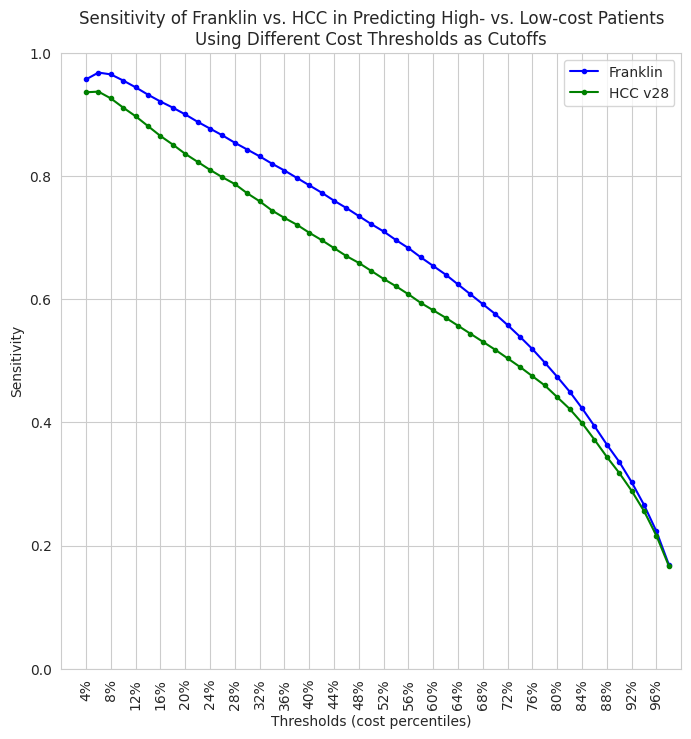

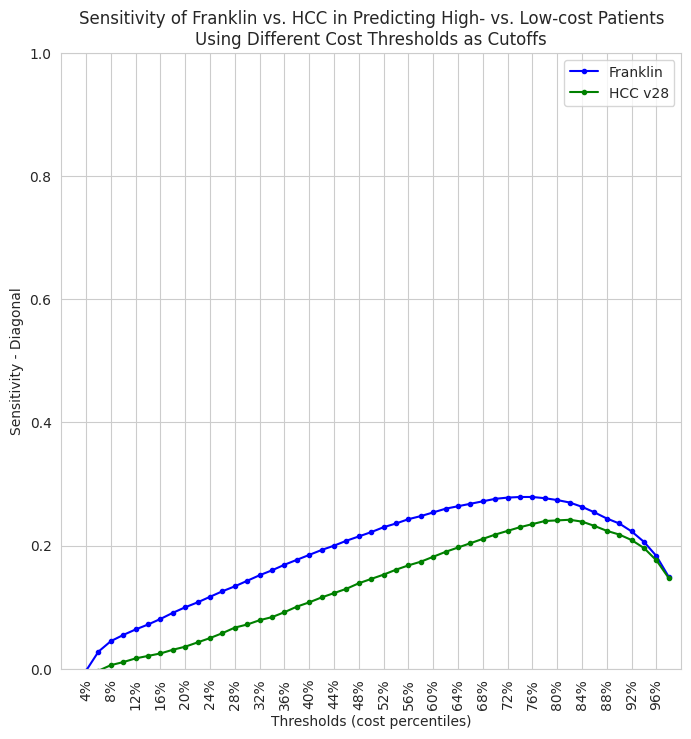


B. Specificity


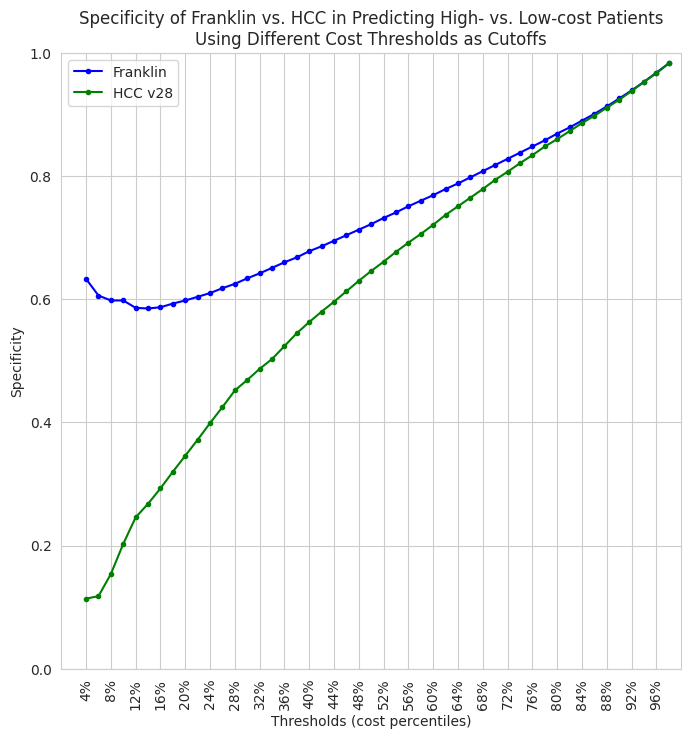

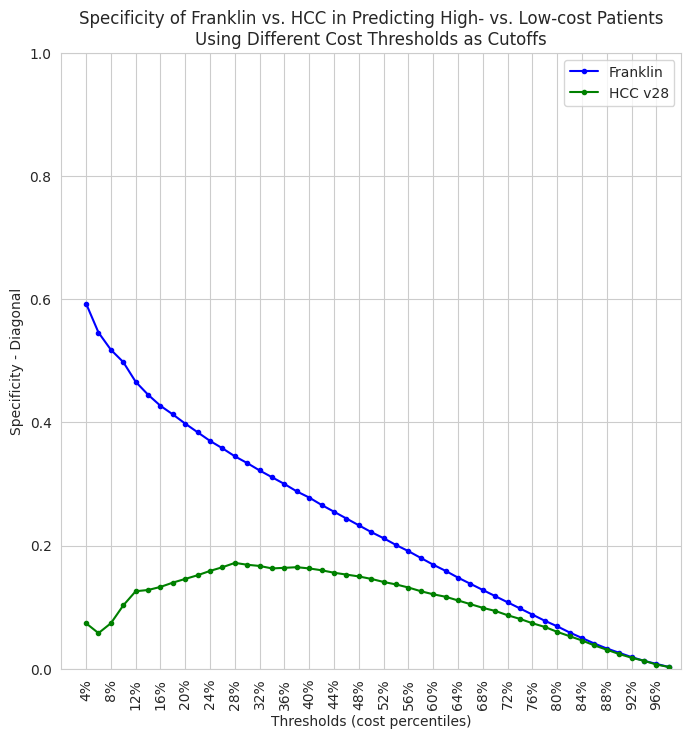


C. Precision


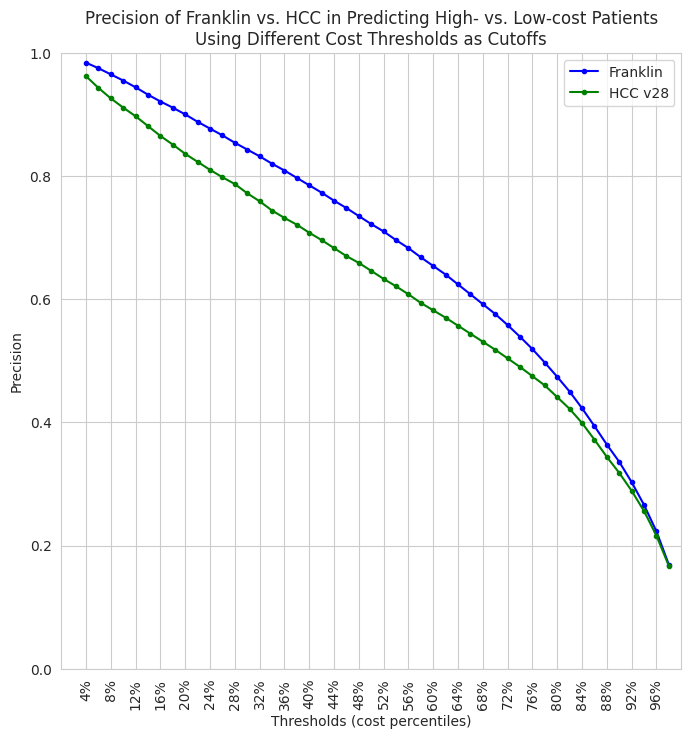

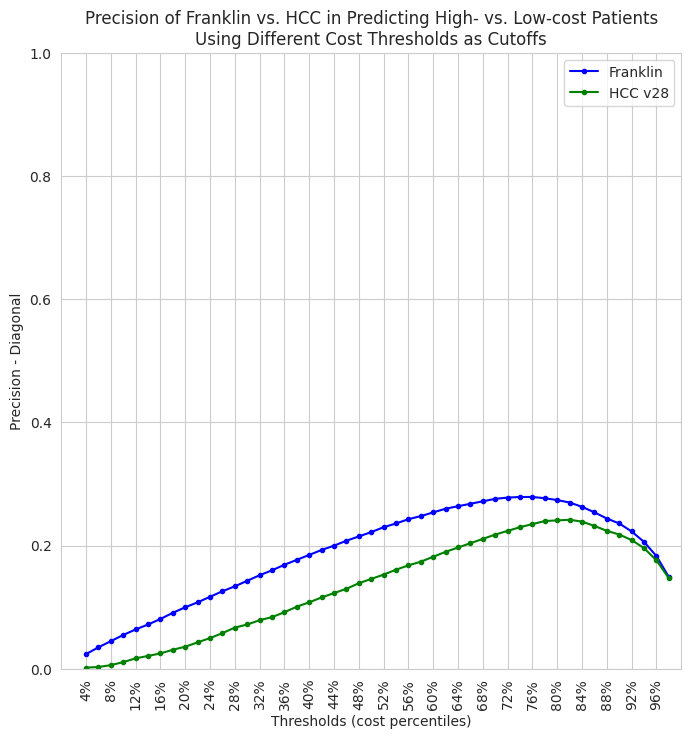


D. NPV


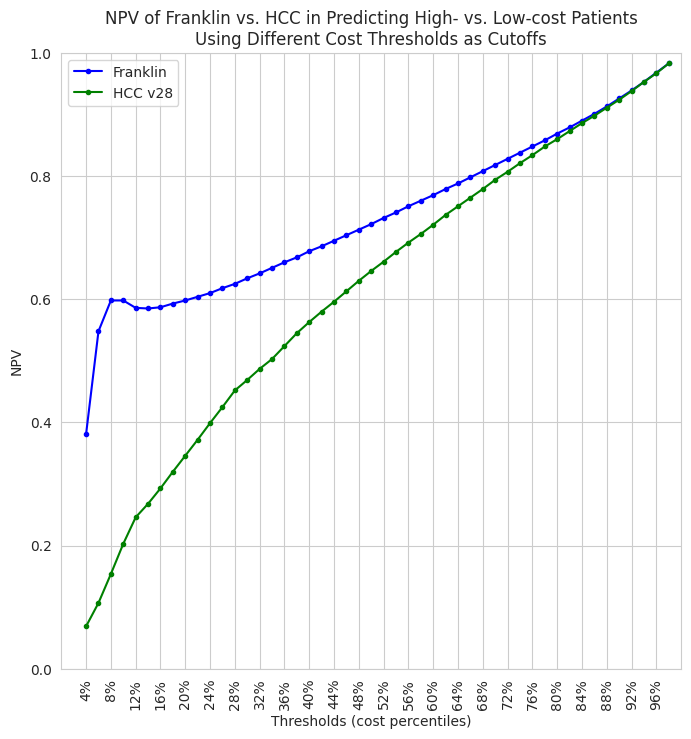

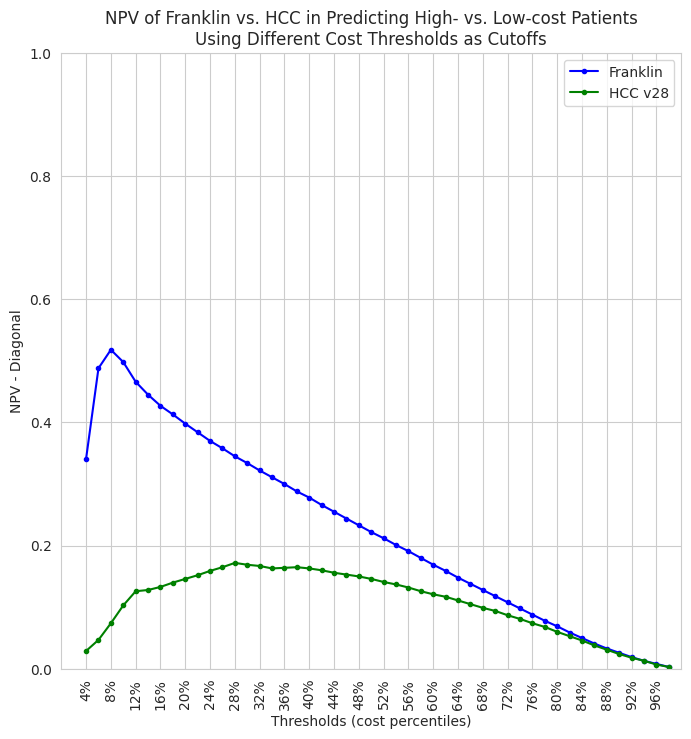


#### D.2.2. Over vs. Underprediction

The prevalence of extreme errors in predictions varies across beneficiaries with 0, 1, or 2+ HCCs can also be considered (eTable D4). Franklin also reduced overprediction of 20 percentiles (20.0% vs. 30.0%) and underprediction of 20 percentiles (18.4% vs. 30.9%). While Franklin performs relatively consistently, it tends towards larger over- and under-predictions as the number of HCCs increases. The prevalence of such mispredictions with Franklin is always less than that of HCC v28, regardless of number of HCCs.

###### eTable D4: Prevalence of extreme over and under predictions with 95% Wilson Binomial proportion confidence intervals.

(A)

| Model Error | Severity | Franklin (% pts) | HCC v28 (% pts) |
| --- | --- | --- | --- |
| Overprediction | predicted percentile > actual percentile + 20 | 19.2 (19.1 – 19.3) | 25.7 (25.6 – 25.8) |
| Overprediction | predicted percentile > actual percentile + 40 | 4.9 (4.9 – 5.0) | 9.9 (9.9 – 10.0) |
| Underprediction | predicted percentile < actual percentile - 20 | 18.2 (18.1 – 18.3) | 24.7 (24.6 – 24.7) |
| Underprediction | predicted percentile < actual percentile - 40 | 7.1 (7.0 – 7.1) | 10.9 (10.9 – 11.0) |

###### eFigure D3: Prevalence of over and under predictions by model (Franklin vs. HCC v28).


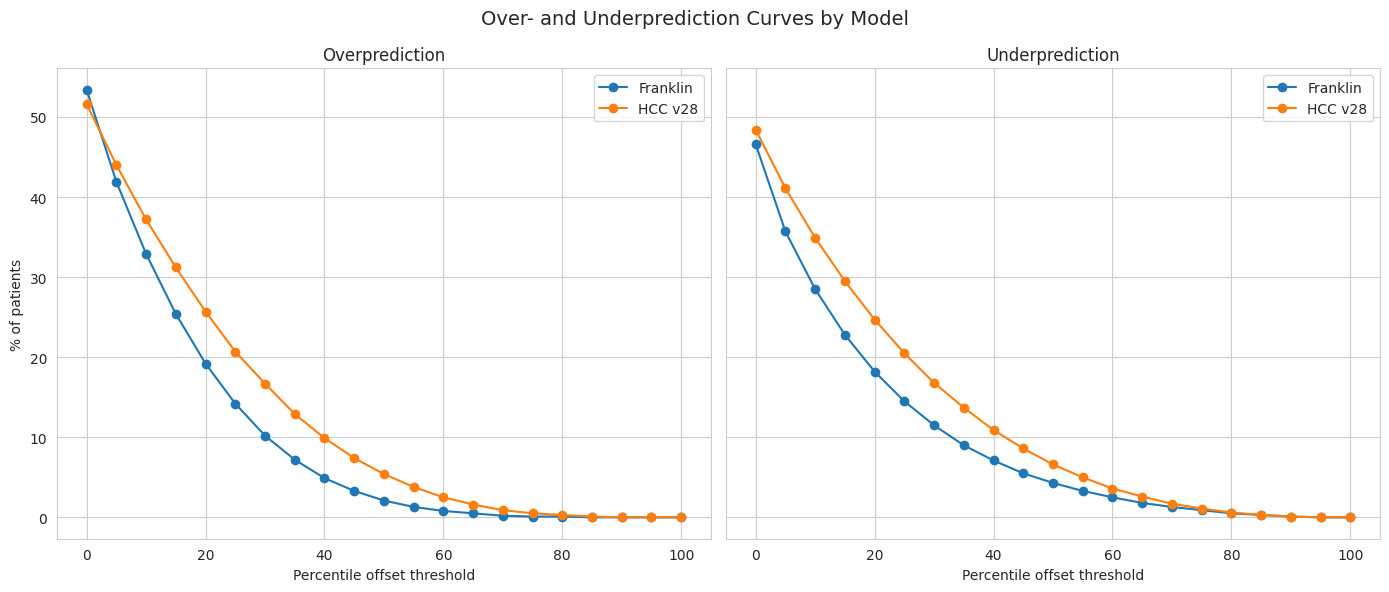


### D.3. Comparison by sociodemographics

#### D.3.1. Model fit

Model performance was compared across various demographic and health cohorts: sex, age, race, geographic region, and health condition.

In general, Franklin maintained a higher performance across all categories than did the HCC model, using both R^2^ and Spearman rho metrics. eTable D5 details the results of both metrics. Franklin was more than 2.5 times more accurate than HCC on the segregated sex models (Franklin R^2^_male_ = 0.46, HCC R^2^_male_ = 0.16; Franklin R^2^_female_ = 0.41, HCC R^2^_female_ = 0.13). Both models performed better on males than on females.

With respect to age, Franklin was once again more accurate than HCC (Franklin R^2^_65-74_ = 0.44, HCC R^2^_65-74_ = 0.13; Franklin R^2^_75-84_ = 0.42, HCC R^2^_75-84_ = 0.14; Franklin R^2^_85+_ = 0.38, HCC R^2^_85+_ = 0.13). Franklin performed significantly better on younger beneficiaries (e.g., ages 65-74, compared to older beneficiaries (e.g., ages 85 and older).

With respect to race and ethnicity, Franklin had a higher performance metrics across each RTI category (Franklin R^2^_White_ = 0.42, HCC R^2^ _White_ = 0.15; Franklin R^2^_Black_ = 0.48, HCC R^2^_Black_ = 0.14; Franklin R^2^_Hispanic_ = 0.55, HCC R^2^_Hispanic_ = 0.09; Franklin R^2^_Asian_ = 0.50, HCC R^2^_Asian_ = 0.10). The Franklin model performed better for non-White beneficiaries compared to White beneficiaries, while the HCC model performed the best for White beneficiaries.

With respect to geographic region, Franklin once again had higher performance metrics across each category (Franklin R^2^_South_ = 0.44, HCC R^2^ _South_ = 0.15; Franklin R^2^_Midwest_= 0.40, HCC R^2^_Midwest_ = 0.15; Franklin R^2^_West_ = 0.46, HCC R^2^_West_ = 0.14; Franklin R^2^_Northeast_ = 0.45, HCC R^2^_Northeast_ = 0.15). Compared to the other regions, Franklin performed less well on the Midwest, while there was little difference in performance across regions in the HCC model.

With respect to population density, Franklin outperformed HCC on all categories (Franklin R^2^_Metropolitan_ = 0.45, HCC R^2^_Metropolitan_ = 0.15; Franklin R^2^_Micropolitan_= 0.39, HCC R^2^_Micropolitan_ = 0.13; Franklin R^2^_Small Town_ = 0.37, HCC R^2^_Small Town_ = 0.12; Franklin R^2^_Rural_ = 0.36, HCC R^2^_Rural_ = 0.11). Both Franklin and HCC performed better in metropolitan and micropolitan areas, compared to small towns and rural areas.

Finally, with respect to several common and/or severe health conditions, Franklin performed better than HCC. Some improvements of Franklin over HCC are noteworthy: while HCC only captures up to 5% of the variance across various cancers, Franklin captures 18-24% of the variance in these subsets. Similarly, while HCC captures 7% of the variance in diabetes, Franklin captures 26% of the variance.

###### eTable D5. Model performance, assessed with Spearman rho and R2, across various population cohorts. 95% confidence intervals evaluated using 1000 bootstrapped samples.

| Subset | % of cohort (n = 851,441) | Franklin Spearman rho | Franklin R2 (log cost) | HCC v28 Spearman rho | HCC v28 R2 (log cost) |
| --- | --- | --- | --- | --- | --- |
| Sex: Female | 55.7 | 0.60 (0.59 - 0.60) | 0.41 (0.41 - 0.42) | 0.40 (0.39 - 0.40) | 0.13 (0.13 - 0.13) |
| Sex: Male | 44.3 | 0.62 (0.62 - 0.63) | 0.46 (0.46 - 0.47) | 0.44 (0.44 - 0.45) | 0.16 (0.16 - 0.16) |
| Age: 65 - 74 | 51.5 | 0.62 (0.62 - 0.62) | 0.44 (0.44 - 0.45) | 0.37 (0.37 - 0.37) | 0.13 (0.13 - 0.13) |
| Age: 75 - 84 | 34.6 | 0.58 (0.58 - 0.59) | 0.42 (0.42 - 0.43) | 0.39 (0.39 - 0.4) | 0.14 (0.14 - 0.14) |
| Age: 85+ | 13.9 | 0.52 (0.51 - 0.52) | 0.38 (0.38 - 0.39) | 0.38 (0.38 - 0.39) | 0.13 (0.12 - 0.13) |
| Original Entitlement: Old age and survivor’s insurance | 92.4 | 0.61 (0.60 - 0.61) | 0.44 (0.44 - 0.44) | 0.41 (0.41 - 0.41) | 0.15 (0.14 - 0.15) |
| Original Entitlement: Disability insurance benefits | 7.6 | 0.63 (0.63 - 0.63) | 0.49 (0.48 - 0.49) | 0.47 (0.47 - 0.48) | 0.20 (0.20 - 0.20) |
| Race: White | 85.7 | 0.60 (0.60 - 0.60) | 0.42 (0.42 - 0.42) | 0.41 (0.41 - 0.41) | 0.15 (0.15 - 0.15) |
| Race: Black/African American | 5.7 | 0.65 (0.64 - 0.65) | 0.48 (0.48 - 0.49) | 0.45 (0.45 - 0.46) | 0.14 (0.14 - 0.15) |
| Race: Unknown | 1.9 | 0.59 (0.59 - 0.60) | 0.41 (0.40 - 0.43) | 0.32 (0.31 - 0.33) | 0.09 (0.09 - 0.10) |
| Race: Asian/Pacific Islander | 2.0 | 0.66 (0.65 - 0.67) | 0.50 (0.49 - 0.52) | 0.39 (0.38 - 0.40) | 0.10 (0.10 - 0.11) |
| Race: Hispanic | 3.5 | 0.71 (0.70 - 0.71) | 0.55 (0.54 - 0.55) | 0.46 (0.45 - 0.46) | 0.09 (0.08 - 0.09) |
| Region: South | 40.6 | 0.61 (0.60 - 0.61) | 0.44 (0.44 - 0.44) | 0.42 (0.42 - 0.42) | 0.15 (0.15 - 0.15) |
| Region: Midwest | 22.7 | 0.59 (0.58 - 0.59) | 0.40 (0.39 - 0.40) | 0.41 (0.41 - 0.41) | 0.15 (0.14 - 0.15) |
| Region: West | 18.7 | 0.63 (0.62 - 0.63) | 0.46 (0.46 - 0.46) | 0.41 (0.41 - 0.41) | 0.14 (0.14 - 0.15) |
| Region: Northeast | 17.6 | 0.61 (0.61 - 0.62) | 0.45 (0.45 - 0.46) | 0.41 (0.41 - 0.42) | 0.15 (0.14 - 0.15) |
| Density: Metropolitan | 76.7 | 0.61 (0.61 - 0.61) | 0.45 (0.45 - 0.45) | 0.42 (0.42 - 0.42) | 0.15 (0.15 - 0.15) |
| Density: Micropolitan | 23.0 | 0.59 (0.59 - 0.59) | 0.39 (0.39 - 0.39) | 0.41 (0.40 - 0.41) | 0.13 (0.13 - 0.13) |
| Density: Small town | 6.4 | 0.58 (0.58 - 0.59) | 0.37 (0.36 - 0.37) | 0.40 (0.40 - 0.41) | 0.12 (0.12 - 0.12) |
| Density: Rural | 4.8 | 0.59 (0.58 - 0.60) | 0.36 (0.36 - 0.38) | 0.40 (0.39 - 0.41) | 0.11 (0.10 - 0.11) |
| Condition: CHF (HCC222, HCC224, HCC225) | 1.7 | 0.43 (0.42 - 0.44) | 0.20 (0.18 - 0.21) | 0.34 (0.33 - 0.34) | 0.09 (0.08 - 0.10) |
| Condition: COPD (HCC280) | 9.6 | 0.51 (0.51 - 0.51) | 0.26 (0.25 - 0.26) | 0.40 (0.39 - 0.40) | 0.12 (0.12 - 0.13) |
| Condition: Diabetes (HCC36, HCC37) | 10.5 | 0.52 (0.52 - 0.52) | 0.26 (0.25 - 0.26) | 0.40 (0.39 - 0.40) | 0.07 (0.07 - 0.08) |
| Condition: CKD (HCC326, HCC327) | 1.4 | 0.47 (0.46 - 0.48) | 0.22 (0.21 - 0.24) | 0.40 (0.39 - 0.41) | 0.13 (0.12 - 0.14) |
| Condition: Metastatic Cancer and Acute Leukemia (HCC17, HCC18, HCC22) | 2.9 | 0.48 (0.47 - 0.49) | 0.22 (0.21 - 0.23) | 0.36 (0.35 - 0.37) | 0.01 (0.00 - 0.02) |
| Condition: Lung and Other Severe Cancers (HCC19, HCC20) | 1.4 | 0.43 (0.43 - 0.44) | 0.18 (0.17 - 0.19) | 0.31 (0.30 - 0.33) | 0.02 (0.01 - 0.02) |
| Condition: Lymphoma and Other Cancers (HCC21, HCC23) | 6.7 | 0.51 (0.50 - 0.51) | 0.24 (0.24 - 0.25) | 0.34 (0.33 - 0.34) | 0.05 (0.04 - 0.05) |
| Condition: Dementia (HCC127) | 3.7 | 0.45 (0.45 - 0.46) | 0.2 (0.19 - 0.21) | 0.34 (0.33 - 0.35) | 0.09 (0.08 - 0.09) |
| Condition: Substance use disorders (HCC135, HCC136, HCC137, HCC138, HCC139) | 1.2 | 0.53 (0.52 - 0.54) | 0.29 (0.27 - 0.30) | 0.40 (0.40 - 0.41) | 0.12 (0.11 - 0.13) |
| Condition: Mental health disorders (HCC151, HCC152, HCC153, HCC154, HCC155) | 3.4 | 0.52 (0.52 - 0.53) | 0.26 (0.26 - 0.27) | 0.36 (0.36 - 0.37) | 0.08 (0.08 - 0.10) |

#### D.3.2. Actual and predicted cost distribution by race

Racial minorities are disproportionately represented in the bottom of the cost distribution. For example, 16% of Black beneficiaries and 23% of Hispanics are in the bottom decile of observed costs, compared to only 9% of white beneficiaries (eTable D6.B). Franklin predictions recover the observed distribution very closely (eTable D6.C), while HCC scores tend to be more evenly distributed due to its inaccuracy in the bottom of the cost distribution (eTable D6.D).

With regards to race, three metrics of fairness were evaluated: demographic parity, calibration gaps, and individual fairness. Overall, the fairness analysis shows minor differences among races, with greater left-shift in Franklin scores and false-negative rates for the top quintile of log costs for Black individuals. These differences need to be explored in future work.

###### eTable D6. Distribution of predicted and actual cost deciles by race, test set: (a) total beneficiary counts by race as recorded in the FFS beneficiary file (b) observed 2019 costs; (c) predicted Franklin costs; (d) predicted HCC costs.

(A)

|  | White | Black | Hispanic | Asian/Pacific islander | Unknown | Other | North American Native |
| --- | --- | --- | --- | --- | --- | --- | --- |
| N | 729,990 | 48,305 | 29,887 | 16,614 | 16,581 | 6,869 | 3,195 |
| % | 85.7% | 5.7% | 3.5% | 2.0% | 1.9% | 0.8% | 0.4% |

(B)

| Cost decile | White | Black | Hispanic | Asian/Pacific islander | Unknown | Other | North American Native |
| --- | --- | --- | --- | --- | --- | --- | --- |
| 1 | 8.8% | 16.0% | 23.1% | 17.0% | 10.5% | 13.5% | 14.8% |
| 2 | 9.8% | 10.8% | 10.6% | 11.9% | 12.2% | 11.4% | 15.9% |
| 3 | 10.0% | 10.0% | 8.8% | 11.4% | 11.9% | 10.2% | 11.7% |
| 4 | 10.0% | 9.7% | 8.6% | 10.7% | 11.1% | 10.4% | 9.3% |
| 5 | 10.1% | 9.3% | 8.3% | 9.9% | 11.1% | 9.8% | 7.9% |
| 6 | 10.2% | 8.8% | 8.1% | 9.3% | 10.4% | 10.6% | 7.9% |
| 7 | 10.2% | 8.5% | 8.6% | 9.0% | 10.0% | 9.2% | 7.4% |
| 8 | 10.2% | 8.5% | 8.6% | 8.0% | 9.3% | 8.7% | 6.3% |
| 9 | 10.3% | 9.0% | 7.8% | 6.5% | 7.5% | 8.2% | 8.8% |
| 10 | 10.3% | 9.4% | 7.6% | 6.4% | 6.0% | 8.1% | 10.0% |

(C)

| Cost decile | White | Black | Hispanic | Asian/Pacific islander | Unknown | Other | North American Native |
| --- | --- | --- | --- | --- | --- | --- | --- |
| 1 | 8.8% | 15.8% | 22.9% | 18.2% | 12.2% | 15.0% | 11.4% |
| 2 | 9.5% | 12.4% | 12.1% | 14.2% | 13.8% | 12.5% | 12.0% |
| 3 | 9.8% | 10.8% | 10.2% | 12.7% | 13.1% | 11.9% | 12.3% |
| 4 | 10.0% | 9.7% | 8.8% | 11.1% | 12.3% | 10.8% | 11.4% |
| 5 | 10.1% | 9.2% | 8.2% | 9.7% | 11.3% | 9.4% | 10.2% |
| 6 | 10.2% | 8.3% | 7.8% | 8.4% | 10.1% | 8.6% | 9.7% |
| 7 | 10.3% | 8.5% | 7.9% | 7.6% | 9.1% | 9.1% | 8.4% |
| 8 | 10.4% | 8.4% | 7.6% | 7.2% | 7.5% | 7.9% | 8.3% |
| 9 | 10.4% | 8.4% | 7.3% | 6.2% | 6.3% | 7.9% | 7.9% |
| 10 | 10.5% | 8.5% | 7.1% | 4.8% | 4.3% | 7.0% | 8.5% |

(D)

| Cost decile | White | Black | Hispanic | Asian/Pacific islander | Unknown | Other | North American Native |
| --- | --- | --- | --- | --- | --- | --- | --- |
| 1 | 9.6% | 10.1% | 12.2% | 13.3% | 20.1% | 8.9% | 9.0% |
| 2 | 9.7% | 9.4% | 11.5% | 12.1% | 19.3% | 10.0% | 8.2% |
| 3 | 9.8% | 9.2% | 11.2% | 11.3% | 17.8% | 10.5% | 8.5% |
| 4 | 9.9% | 10.8% | 11.2% | 12.6% | 5.4% | 13.7% | 10.7% |
| 5 | 9.9% | 11.0% | 11.2% | 12.2% | 7.6% | 11.9% | 11.9% |
| 6 | 10.0% | 10.4% | 9.9% | 10.0% | 7.8% | 10.9% | 10.4% |
| 7 | 10.1% | 10.5% | 8.7% | 8.4% | 6.5% | 9.6% | 10.9% |
| 8 | 10.2% | 9.6% | 8.5% | 7.6% | 6.2% | 8.8% | 9.8% |
| 9 | 10.4% | 9.1% | 7.7% | 6.4% | 4.5% | 7.7% | 10.2% |
| 10 | 10.3% | 9.9% | 7.8% | 6.0% | 4.7% | 7.9% | 10.4% |

eFigure D4. Demographic parity: distribution of Franklin scores for Black and White beneficiaries


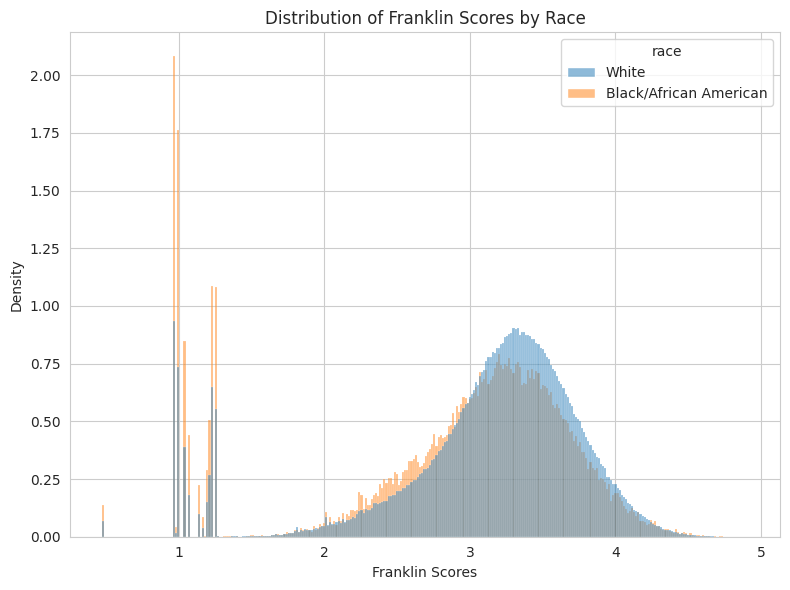


eFigure D5. Calibration gaps: predicted vs. actual log cost bins, by race

Each point represents the average observed vs. predicted decile of log cost. Overall, Franklin is well calibrated above the second decile of log costs. At the lowest end of the cost spectrum, there are some calibration gaps among race groups. When put in dollar amounts, the highest cost in each bins are 1: $183.50, 2: $551.08, 3: $921.57, 4: $1,308.18, 5: $1,744.82, 6: $2,279.34, 7: $2,998.16, 8: $4,101.04, 9: $6,308.57, 10: $81,282.05.


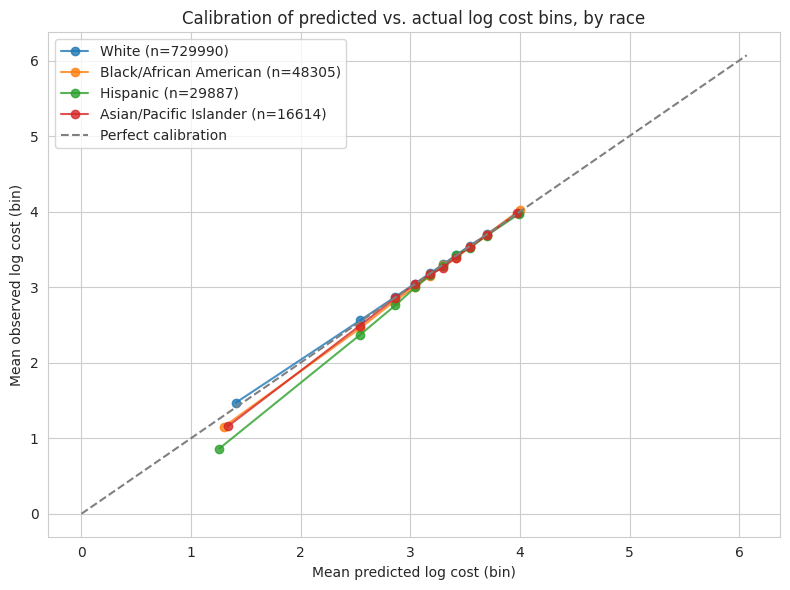


eTable D7. Individual fairness: FNR for predicting the top quintile of log cost, by race

| Race | False Negative Rates |
| --- | --- |
| Asian/Pacific Islander | 0.621 |
| Black/African American | 0.557 |
| Hispanic | 0.573 |
| White | 0.518 |

#### D.3.3. Decedent analysis.

Decedents were excluded from the training data and from model evaluation, as accounting for mortality would require statistical modeling choices that are distinct from demonstrating the value of ML for improving risk adjustment. Specifically, the time of death within a calendar year impacts the cost incurred in that year, leading to potential bias in model evaluation. In addition, hospice claims were not available to us, reducing the accuracy of cost capture.

Nevertheless, given a cost prediction model it is possible to evaluate its accuracy on the decedent population. We evaluated the performance of Franklin and HCC on beneficiaries in the test set who expired in the 2019 (the prediction year) but otherwise met all the inclusion and exclusion criteria (the requirement for 24 months of continuous FFS coverage was relaxed accordingly, though decedents with MA coverage were still excluded). This cohort included n=34,566 decedents.

Both HCC and Franklin are inaccurate when evaluated on decedents: R^2^ log cost Franklin 0.027 (CI 0.012-0.035), HCC 0.021 (0.018-0.025, difference not statistically significant), reflecting the difficulty of predicting decedent costs without a mortality model. Franklin remains more accurate than HCC in terms of ranking: Spearman rho Franklin 0.247 (CI 0.236-0.262), HCC 0.159 (CI 0.151-0.163, P<0.001).

Franklin remains significantly more accurate than HCC when augmenting the original test cohort by the cohort of decedents (n=886,007 total): R^2^ log cost Franklin 0.421 (CI 0.418-0.422), HCC 0.151 (0.149-0.152, P<0.001), Spearman rho Franklin 0.600 (CI 0.599-0.601), HCC 0.425 (CI 0.425-0.426, P<0.001).

### D.4. Model calibration and metrics of actuarial risk.

Franklin predictions were calibrated to achieve predictive ratios (PR) close to 1 in each risk decile (Table 3 in the main manuscript). IQR and decile ranges of observed costs in each risk decile were calculated for Franklin and HCC (Table 3 in the article and eTable D7). Confidence intervals were estimated using 10,000 bootstrapped samples within each decile.

###### eTable D8. (A) IQR and (B) decile range for observed costs in each decile of Franklin and HCC, and the ratio between them.

(A)

|  | Franklin | | | HCC | | | Franklin / HCC Ratio | | |
| --- | --- | --- | --- | --- | --- | --- | --- | --- | --- |
| Cost decile | Value ($) | 95% CI  Low ($) | 95% CI  High ($) | Value ($) | 95% CI  Low ($) | 95% CI  High ($) | Value (%) | 95% CI Low (%) | 95% CI High (%) |
| 1 | 437 | 406 | 471 | 1,881 | 1,799 | 1,963 | 23.3 | 21.3 | 25.4 |
| 2 | 1,180 | 1,122 | 1,244 | 2,153 | 2,057 | 2,246 | 54.8 | 54.1 | 58.5 |
| 3 | 1,609 | 1,536 | 1,686 | 2,319 | 2,052 | 2,233 | 75.2 | 70.9 | 79.8 |
| 4 | 1,996 | 1,899 | 2,091 | 2,587 | 2,470 | 2,696 | 77.1 | 72.5 | 82.1 |
| 5 | 2,461 | 2,346 | 2,586 | 2,927 | 2,803 | 3,062 | 84.1 | 79.0 | 89.3 |
| 6 | 3,073 | 2,938 | 3,226 | 3,514 | 3,341 | 3,687 | 87.4 | 82.2 | 93.3 |
| 7 | 3,989 | 3,787 | 4,216 | 4,442 | 4,211 | 4,692 | 89.8 | 83.9 | 96.5 |
| 8 | 5,592 | 5,271 | 5,948 | 6,526 | 6,128 | 6,936 | 85.7 | 79.3 | 92.8 |
| 9 | 9,122 | 8,622 | 9,749 | 10,682 | 10,032 | 11,309 | 85.4 | 79.6 | 92.4 |
| 10 | 21,721 | 20,694 | 22,677 | 22,131 | 21,118 | 23,281 | 98.1 | 94.0 | 101.9 |

(B)

|  | Franklin | | | HCC | | | Franklin / HCC Ratio | | |
| --- | --- | --- | --- | --- | --- | --- | --- | --- | --- |
| Cost decile | Value ($) | 95% CI  Low ($) | 95% CI  High ($) | Value ($) | 95% CI  Low ($) | 95% CI  High ($) | Value (%) | 95% CI Low (%) | 95% CI High (%) |
| 1 | 2,029 | 1,831 | 2,248 | 4,907 | 4,635 | 5,204 | 41.3 | 36.8 | 46.2 |
| 2 | 4,076 | 3,763 | 4,439 | 5,590 | 5,274 | 5,914 | 72.9 | 66.4 | 80.3 |
| 3 | 5,443 | 5,003 | 5,971 | 5,761 | 5,394 | 6,206 | 94.5 | 85.0 | 104.6 |
| 4 | 6,908 | 6,298 | 7,532 | 7,688 | 7,095 | 8,299 | 89.8 | 80.5 | 100.5 |
| 5 | 8,857 | 8,077 | 9,613 | 9,731 | 8,962 | 10,667 | 91.0 | 80.6 | 101.6 |
| 6 | 11,861 | 10,826 | 12,936 | 12,486 | 11,469 | 13,405 | 95.0 | 85.3 | 106.3 |
| 7 | 16,032 | 14,961 | 17,185 | 16,354 | 15,321 | 17,547 | 98.0 | 89.6 | 107.0 |
| 8 | 20,968 | 19,761 | 22,158 | 22,507 | 21,178 | 23,818 | 93.2 | 86.3 | 100.2 |
| 9 | 28,836 | 27,400 | 30,360 | 30,809 | 29,357 | 32,376 | 93.6 | 87.9 | 99.6 |
| 10 | 51,897 | 49,690 | 53,957 | 53,648 | 51,378 | 55,897 | 96.7 | 93.6 | 99.6 |

### D.5. Quantifying contribution of individual features to Franklin Scores.

SHapley Additive exPlanations values^6^ can be used to quantify the contribution of individual features to the XGBoost model (eFigure D4).

eFigure D6. SHAP plot of the tuned XGBoost model. The central line at 0 indicates no importance to the outcome. Points to the left of the center indicate lower cost, whereas points to the right of the center indicate higher cost. The colors of the plot represent feature values, where red indicates higher feature values and blue represents lower feature values. Recall that the features represent distances between a person’s ICD embeddings and cluster centroids. Low feature values (blue color) represent a high similarity (small distances) between the embeddings and centroids. For example, the blue line to the right of 0 under “cancer (e.g., colon, lung) / side effects” indicates that people with cancer-related ICDs are associated with higher costs. Features are plotted in the order of average importance.


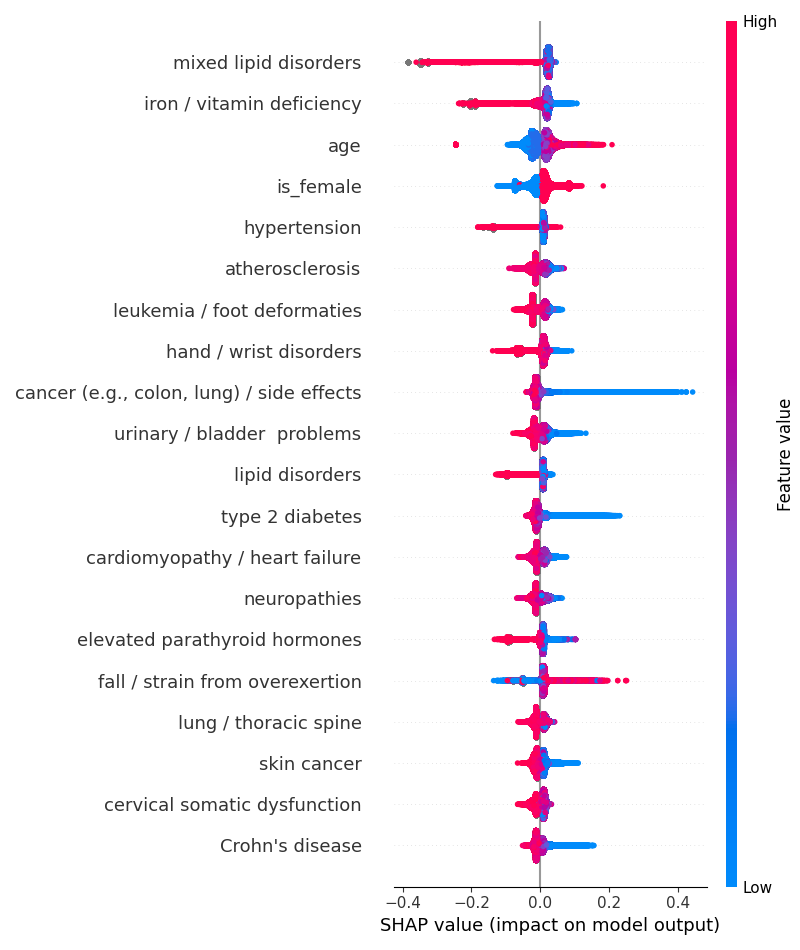


# References

1. Welcome | The Tuva Project. Accessed December 17, 2024. https://www.thetuvaproject.com/

2. 2023 ANNUAL REPORT OF THE BOARDS OF TRUSTEES OF THE FEDERAL HOSPITAL INSURANCE AND FEDERAL SUPPLEMENTARY MEDICAL INSURANCE TRUST FUNDS. Published online March 31, 2023. https://www.cms.gov/oact/tr/2023

3. Mikolov T, Sutskever I, Chen K, Corrado G, Dean J. Distributed Representations of Words and Phrases and their Compositionality. *arXiv*. Preprint posted online October 16, 2013. doi:10.48550/arXiv.1310.4546

4. Chen T, Guestrin C. XGBoost: A Scalable Tree Boosting System. In: *Proceedings of the 22nd ACM SIGKDD International Conference on Knowledge Discovery and Data Mining*. KDD ’16. Association for Computing Machinery; 2016:785-794. doi:10.1145/2939672.2939785

5. Duan N. Smearing Estimate: A Nonparametric Retransformation Method. *Journal of the American Statistical Association*. 1983;78(383):605-610. doi:10.2307/2288126

6. Lundberg SM, Lee SI. A Unified Approach to Interpreting Model Predictions. In: *Advances in Neural Information Processing Systems*. Vol 30. Curran Associates, Inc.; 2017. Accessed December 17, 2024. https://proceedings.neurips.cc/paper/2017/hash/8a20a8621978632d76c43dfd28b67767-Abstract.html
